# Supplementary material for: Measurements of the Timescale and Conformational Space of AMPA Receptor Desensitization
Source: Biophys J. 2020 Jun 4;119(1):206–18. doi: 10.1016/j.bpj.2020.05.029 (PMC7335938; doi:10.1016/j.bpj.2020.05.029)
Supplement: Document S1. Supporting Materials and Methods, Figs. S1–S2, and Tables S1–S2 [file mmc1.pdf]

Biophysical Journal, Volume *119*

Supplemental Information

Measurements of the Timescale and Conformational Space of AMPA Receptor Desensitization

Hector Salazar, Sabrina Mischke, and Andrew J.R. Plested

Supplementary Table 1 Hodgkin-Huxley type fits to GluA2 recovery from desensitization with 1, 2 or 3 components.

Figure	Function	Duration of conditioning pulse	Goodness of fit (χ^2)	Parameters									
				y_0	a_1	a_2	k_1	k_2	k_3	m_1	m_2	m_3	y_{max}
S1A	H.-H. 1 component	50ms	44	0.000 ± 0.004			43 ± 1			*2*			1.022 ± 0.003
		200ms	36	0.000 ± 0.006			44 ± 1			*2*			1.022 ± 0.004
		800ms	41	0.000 ± 0.006			44 ± 1			*2*			1.007 ± 0.004
		5s	153	0.000 ± 0.002			34 ± 1			*2*			0.983 ± 0.006
S1B	H.-H. 1 component	50ms	13	0.010 ± 0.006			54 ± 3			2.8 ± 0.3			1.017 ± 0.003
		200ms	14	0.0011 ± 0.009			52 ± 3			2.6 ± 0.3			1.018 ± 0.004
		800ms	18	0.00 ± 0.01			52 ± 3			2.5 ± 0.3			1.001 ± 0.004
		5s	29	0.002 ± 0.006			46 ± 2			2.8 ± 0.2			0.967 ± 0.006
S1C	H.-H. 2 component	50ms	940	0.000 ± 0.009	0.5 ± 6000		21 ± 1000	21 ± 353		*1*	*1*		1.035 ± 0.004
		200ms	610	0.00 ± 0.01	0.6 ± 8000		24 ± 1000	24 ± 2000		*1*	*1*		1.033 ± 0.005
		800ms	610	0.00 ± 0.01	0.7 ± 6000		24 ± 1000	24 ± 3000		*1*	*1*		1.020 ± 0.005
		5s	2079	0.000 ± 0.006	0.5 ± 6000		12 ± 4000	12 ± 3000		*1*	*1*		1.02 ± 0.02
S1D	H.-H. 2 component	50ms	44	0.000 ± 0.006	0.79 ± 3000		43 ± 2000	42 ± 6000		*2*	*2*		1.022 ± 0.004
		200ms	36	0.000 ± 0.008	0.50 ± 8000		44 ± 8000	45 ± 7000		*2*	*2*		1.022 ± 0.004
		800ms	42	0.000 ± 0.009	1 ± 5		43 ± 38	30 ± 4000		*2*	*2*		1.006 ± 0.005
		5s	154	0.000 ± 0.005	0.6 ± 6000		34 ± 4000	33 ± 5000		*2*	*2*		0.984 ± 0.008
S1E	H.-H. 2 component	50ms	9	0.01 ± 0.005	0.9 ± 0.2		61 ± 4	20 ± 20		*3*	*2*		1.02 ± 0.004
		200ms	4	0.005 ± 0.006	0.96 ± 0.03		61 ± 3	10 ± 5		*3*	*2*		1.025 ± 0.006
		800ms	8	0.005 ± 0.006	0.91 ± 0.07		63 ± 4	15 ± 10		*3*	*2*		1.007 ± 0.004
		5s	14	0.0002 ± 0.004	0.84 ± 0.07		55 ± 3	14 ± 6		*3*	*2*		0.978 ± 0.007
S1F	H.-H. 3 component	50ms	39	0.017 ± 0.007	0.79 ± 0.19	0.2 ± 530	75 ± 5	30 ± 30000	60 ± 2000	*4*	*2*	*2*	
		200ms	34	0.01 ± 0.01	0.78 ± 0.25	0.1 ± 440	75 ± 13	33 ± 4000	31 ± 5000	*4*	*2*	*2*	
		800ms	6	0.016 ± 0.006	0.85 ± 0.08	0.16 ± 0.07	76 ± 4	16 ± 10	3 ± 3	*4*	*2*	*2*	
		5s	7	0.008 ± 0.005	0.69 ± 0.09	0.28 ± 0.09	71 ± 4	20 ± 6	1 ± 2	*4*	*2*	*2*	
5	H.-H. 2 component	50ms	6	0.015 ± 0.006	0.7 ± 0.2		76 ± 4	30 ± 8		*4*	*2*		1.020 ± 0.004
		200ms	3	0.016 ± 0.005	0.91 ± 0.04		73 ± 3	13 ± 5		*4*	*2*		1.024 ± 0.005
		800ms	7	0.015 ± 0.007	0.8 ± 0.1		77 ± 5	22 ± 9		*4*	*2*		1.007 ± 0.005
		5s	7	0.008 ± 0.004	0.70 ± 0.08		71 ± 4	19 ± 4		*4*	*2*		0.978 ± 0.007

Parameters are shown ± their standard deviation from the fit in IGOR Pro. Parameters in red are not properly determined, and those italicised were fixed. The goodness of fit (χ^2) is coloured from worst (red) to best (green). The two component H.-H. fit with exponents m_1 and m_2 fixed to 1 is equivalent to a double exponential fit. The 1 and 2 component H.-H. functions are given in the methods, main text . The H.-H. 3 Component function (maximum fixed to 1) is as follows:

$$f(t) = y_0 + a_1 \cdot (1 - exp(-k_1 t))^{m_1} + a_2 \cdot (1 - exp(-k_2 t))^{m_2} + (1 - a_1 - a_2 - y_0) \cdot (1 - exp(-k_3 t))^{m_3}$$

Supplementary Table 2 Sigmoid and Hill type fits to GluA2 recovery from desensitization

Figure	Function	Duration of conditioning pulse	Goodness of fit (χ^2)	Parameters				
				y_0	τ	m	y_{max}	t_{50}
S2A	Sigmoid	50ms	100	0.00 ± 0.01	0.0083 ± 0.0006		1.01 ± 0.01	0.0314 ± 0.0008
		200ms	104	0.00 ± 0.02	0.0081 ± 0.0007		1.01 ± 0.02	0.0305 ± 0.0009
		800ms	111	0.00 ± 0.02	0.0089 ± 0.0007		0.99 ± 0.02	0.0313 ± 0.0009
		5s	157	0.00 ± 0.01	0.0095 ± 0.0006		0.95 ± 0.01	0.0381 ± 0.0009
S2B	Hill	50ms	9	0.014 ± 0.004		2.5 ± 0.1	1.022 ± 0.004	0.0269 ± 0.0007
		200ms	5	0.009 ± 0.007		2.5 ± 0.1	1.022 ± 0.004	0.0265 ± 0.0009
		800ms	8	0.008 ± 0.008		2.4 ± 0.1	1.007 ± 0.004	0.0265 ± 0.0008
		5s	13	0.002 ± 0.005		2.4 ± 0.1	0.975 ± 0.006	0.0317 ± 0.0008

Parameters are shown ± their standard deviations from fits in IGOR Pro. The parameter y_0 was constrained to be greater than zero for the sigmoid fits. The goodness of fit (χ^2) is coloured from worst (red) to best (green) according to the scale in Supplementary Table 1.

Sigmoid function:

$$f(t) = y_0 + \left(y_{max} \middle/ \left(1 + exp\left(\frac{t_{50} - t}{\tau}\right)\right)\right)$$

Hill equation:

$$f(t) = y_0 + (y_{max} - y_0) \,/\, \left(1 + \left(\frac{t_{50}}{t}\right)^m\right)$$

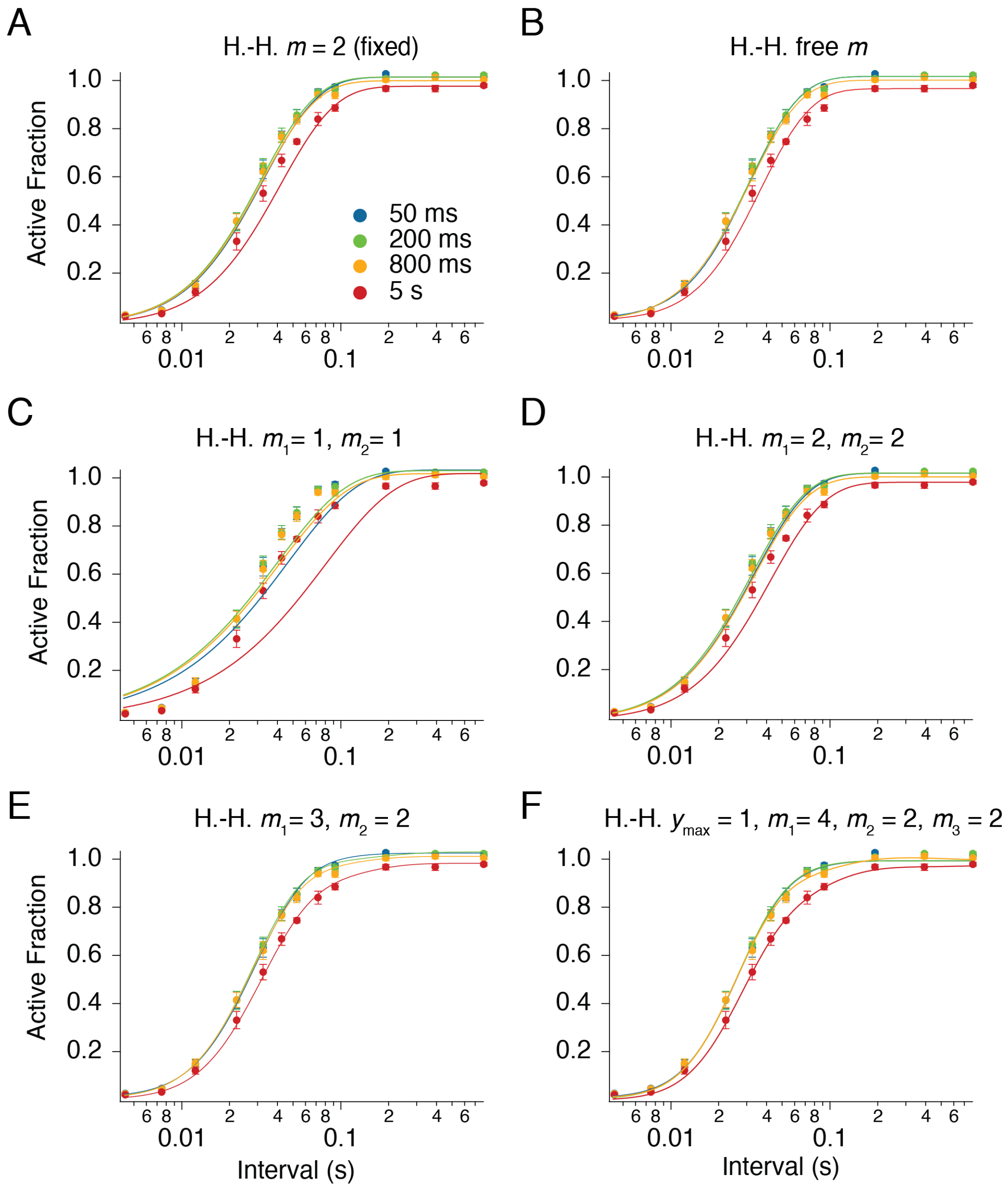

Supplementary Figure 1 Hodgkin-Huxley fits to GluA2 recovery from desensitization

Parameters and χ^2 values for each fitted curve are given in Supplementary Table 1. Data points from Figure 5.

(A) Single component H.-H. fit with slope fixed to 2 cannot describe the recovery following a 5 s pulse (red). The early phase of recovery is poorly captured by this function because it is not steep enough.

(B) Allowing the slope to vary in a single component H.-H. function makes a better description of the early phase, but not for the recovery following the 5 s conditioning pulse (red curve). The disadvantage of a free slope is that rates and slopes are strongly negatively correlated during the fitting process.

(C) A two component H.-H. function with slopes fixed to 1 (equivalent to a double exponential) is a very poor descriptor, because the slopes are too shallow. Parameters in this fit are undetermined.

(D) A two component H.-H. function with both slopes fixed to two gives a poor description of the data following a 5 s conditioning pulse. For the shorter pulse data, the parameters are not determined.

(E) With one slope fixed to 3 and one slope fixed to 2, the description is good and all parameters are well determined. The goodness of fit (χ^2) for each curve was similar to fits with $m_1 = 4$ and $m_2 = 2$ (Figure 5), except for recovery following the 5 s pulse, where the fit is definitively worse ($\chi^2 = 14$ vs. 7).

(F) The H.-H. function with three components (with fixed slopes, and maximum fixed to 1) gives a good description for recovery following the 5 s condition pulse. For shorter conditioning pulses, several parameters are not defined. For the recovery following the 800 ms pulse (orange curve), the slowest component ($k_3 = 3$ s^{-1}) has a negative amplitude.

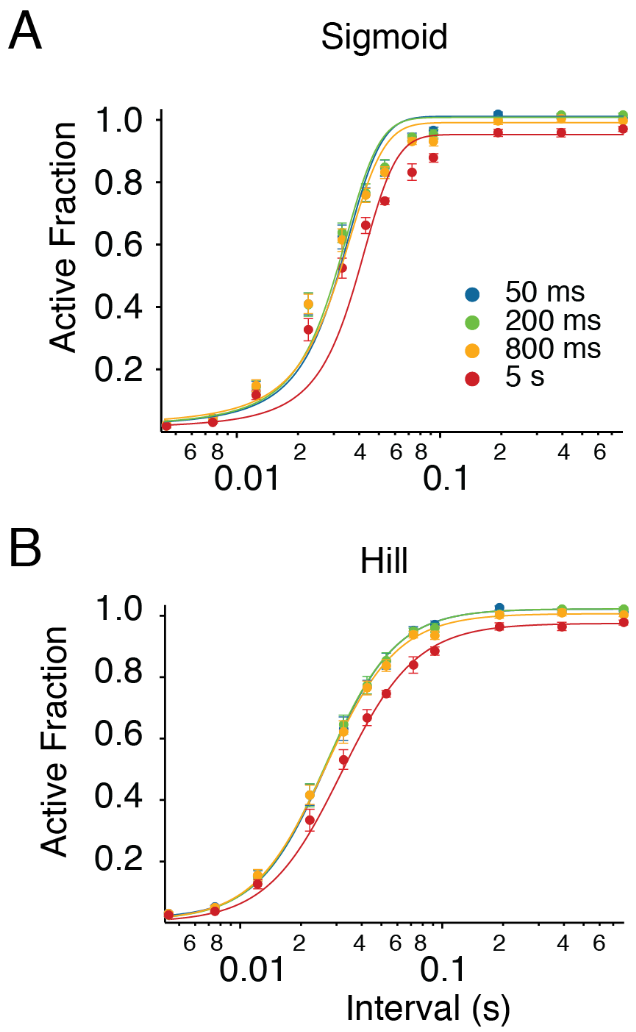

Supplementary Figure 2 Sigmoid and Hill fits to GluA2 recovery from desensitization
Equations, and parameters and χ^2 values for each fitted curve are given in Supplementary Table 2 and its legend.
(A) Fits of sigmoid curves are extremely poor at describing the recovery, because they are too steep. The y_0 parameter was fixed to be positive.
(B) Allowing a free slope in the Hill equation describes the recovery from desensitization data surprisingly well, almost as well as any other function. The slope of the early phase of recovery is poorly described for the recovery after the 5 s conditioning pulse (red curve). The disadvantage of a free slope is that rate and slope are strongly negatively correlated during the fitting process. Overall, fitting the data with the Hill relation is problematic because the physical meaning of the equation with relation to conformational changes over time is unclear.
