# Supplementary material for: Measurements of the Timescale and Conformational Space of AMPA Receptor Desensitization
Source: Biophys J. 2020 Jun 4;119(1):206–18. doi: 10.1016/j.bpj.2020.05.029 (PMC7335938; doi:10.1016/j.bpj.2020.05.029)
Supplement: Document S2. Article plus Supporting Material [file mmc2.pdf]

Biophysical Journal
Article

Biophysical Society

Measurements of the Timescale and Conformational Space of AMPA Receptor Desensitization

Hector Salazar,[1,2,3] Sabrina Mischke,[1,2,3] and Andrew J. R. Plested[1,2,3,*]
[1]Institute of Biology, Cellular Biophysics, Humboldt Universität zu Berlin, Berlin, Germany; [2]Leibniz-Forschungsinstitut für Molekulare Pharmakologie Berlin, Germany; and [3]Charité – Universitätsmedizin Berlin, corporate member of Freie Universität Berlin and Humboldt Universität zu Berlin, and Berlin Institute of Health, NeuroCure Cluster of Excellence, Berlin, Germany

ABSTRACT Ionotropic glutamate receptors are ligand-gated ion channels that mediate excitatory synaptic transmission in the central nervous system. Desensitization of the α-amino-3-hydroxy-5-methyl-4-isoxazolepropionic acid subtype after glutamate binding appears critical for brain function and involves rearrangement of the ligand binding domains (LBDs). Recently, several full-length structures of ionotropic glutamate receptors in putative desensitized states were published. These structures indicate movements of the LBDs that might be trapped by cysteine cross-links and metal bridges. We found that cysteine mutants at the interface between subunits A and C and lateral zinc bridges (between subunits C and D or A and B) can trap freely desensitizing receptors in a spectrum of states with different stabilities. Consistent with a close approach of subunits during desensitization processes, the introduction of bulky amino acids at the A-C interface produced a receptor with slow recovery from desensitization. Further, in wild-type GluA2 receptors, we detected the population of a stable desensitized state with a lifetime around 1 s. Using mutations that progressively stabilize deep desensitized states (E713T and Y768R), we were able to selectively protect receptors from cross-links at both the diagonal and lateral interfaces. Ultrafast perfusion enabled us to perform chemical modification in less than 10 ms, reporting movements associated to desensitization on this timescale within LBD dimers in resting receptors. These observations suggest that small disruptions of quaternary structure are sufficient for fast desensitization and that substantial rearrangements likely correspond to stable desensitized states that are adopted relatively slowly on a timescale much longer than physiological receptor activation.

SIGNIFICANCE α-Amino-3-hydroxy-5-methyl-4-isoxazolepropionic acid (AMPA)-type glutamate receptors are central components of fast synaptic transmission in the brain. Desensitization occurs as a natural consequence of AMPA receptor activation and can reduce the response of a synapse. AMPA receptor desensitization is also necessary for brain development. Molecular structures of AMPA receptors in putative desensitized states predict wide-ranging movements during desensitization. Here, we performed cross-linking experiments on mutant receptors that we subjected to desensitizing conditions over time periods from milliseconds to minutes. These experiments allowed us to count desensitized configurations and rank them according to their stabilities. These data show that large-scale rearrangements occur during long glutamate exposures that are probably not seen in a healthy brain, whereas smaller changes in structure probably suffice for any desensitization at synapses.

INTRODUCTION

Glutamate receptor ion channels mediate most of the fast excitatory synaptic transmission in the vertebrate central nervous system (1). Glutamate binding initiates the opening of an integral ion pore, permitting cations to flow into the postsynaptic cell. The α-amino-3-hydroxy-5-methyl-4-isoxazolepropionic acid (AMPA) receptor subtype desensitizes rapidly and profoundly in response to the sustained presence of glutamate for more than ~25 ms (2,3). The number of receptors available to respond to glutamate is consequently reduced during a phase of recovery from desensitization, which in turn can determine the amplitude of postsynaptic responses (4,5). The timescale of recovery from desensitization, being for AMPA receptors in the order of tens to hundreds of milliseconds, is pertinent during high-frequency release of glutamate (above 10 Hz). Steady-state desensitization may offer protection during pathological glutamate insults that lead to brain damage (6) and during development (7). Finally, desensitized-like conformations might

Submitted November 20, 2019, and accepted for publication May 29, 2020.
*Correspondence: andrew.plested@hu-berlin.de
Editor: Philip Biggin.
https://doi.org/10.1016/j.bpj.2020.05.029
© 2020 Biophysical Society.
This is an open access article under the CC BY license (http://creativecommons.org/licenses/by/4.0/).

be important during biogenesis, trafficking, or in general, any cellular situation in which activation would be problematic. A connection between surface expression and desensitization has been shown for AMPA and kainate receptors (8,9). These observations provide motivation for understanding the molecular basis of AMPA receptor desensitization.

AMPA receptors assemble from four subunits, each comprising an extracellular amino-terminal domain, a ligand binding domain (LBD) that is connected to the ion channel forming transmembrane domain and a C-terminal domain. The LBD is formed from an upper D1 and a lower D2 lobe. Upon the binding of glutamate, the LBD closes. This motion provokes the separation of the D2 domains, leading to the opening of the receptor gate (10). After activation, the receptor transits to desensitized states in ~10 ms, at least in part because of dissociation of the dimer interface formed by the D1 domains (11).

Recent structures of the full-length AMPA receptor in putative desensitized states suggest that a wide conformational space is sampled. Initial cryo-electron microscopy (cryo-EM) structures of the GluA2 receptor in desensitizing conditions showed a set of three-dimensional classes in which the extracellular domains were progressively spread out (12). A 5-fluorowillardiine-bound structure of GluA2 (13) also showed a large rearrangement of the amino-terminal domain and LBDs. Structures of the related GluK2 kainate receptor show that the LBDs adopt a fourfold symmetric arrangement (14) with individual subunits rotating by more than 120° from their active state dimer positions (Fig. 1; (15)). More recent desensitized state structures of GluA2 in the presence of the accessory proteins GSGL1L or TARP γ-2 revealed compact desensitized arrangements, with the LBD dimers losing their internal twofold rotational symmetry (16,17).

We previously used cysteine and metal-bridge cross-linking to identify compact arrangements of the LBD tetramer associated with the activation of the AMPA receptor. These include the "closed angle" conformation (18), multiple compact forms for LBDs fully bound to glutamate (19), and the partial agonist 5-fluorowillardiine (20). The latter assembly featured a parallel shift of the individual dimers. In this previous work, we largely used cyclothiazide (CTZ) to prevent desensitized arrangements of the LBD layer. More recently, we used bifunctional cysteine cross-linkers to measure the extent to which the LBD tetramer opens up in both active and desensitized states (21).

In this study, we revisited our earlier observation that desensitized receptors can be cross-linked very stably between A and C subunits by the A665C disulfide bond (18). Using a fast perfusion system, we used several strategies on mutant and wild-type (WT) receptors to count conformational states attained during desensitization. Distinct from our previous work in which we trapped active states, here, we employed conditions to enrich desensitized states and also examined the inactivation of apo receptors. Comparing potential desensitized states obtained in crystallographic and cryo-EM to the geometric constraints imposed by disulfide bonds and metal bridges suggests that compact desensitized arrangements can best account for desensitization on the physiological timescale.

MATERIALS AND METHODS

Electrophysiology

All mutants were generated on the rat GluA2flip background by overlap PCR and confirmed by double-stranded DNA sequencing. For consistency with previous reports, the numbering of mutated amino acids assumes a 21-residue signal peptide for GluA2. WT and mutant AMPA receptors were expressed transiently in HEK-293 cells for outside-out patch recording. All patches were voltage clamped between −30 and −60 mV. Currents were filtered at 1–10 kHz (−3 dB cutoff, 8-pole Bessel) and recorded using AxoGraph X (AxoGraph Scientific) via an InstruTECH ITC-18 interface (HEKA Elektronik) at a 20-kHz sampling rate.

The external solution in all experiments contained 150 mM NaCl, 0.1 mM $MgCl_2$, 0.1 mM $CaCl_2$, and 5 mM HEPES, titrated to pH 7.3 with NaOH, to which we added drugs, agonists, redox agents, zinc, and ion chelators. CTZ stock solution was prepared in DMSO and added at 100 μM to the external solution. Drugs were obtained from Tocris Bioscience (Bristol, UK), Ascent Scientific (Bristol, UK), or Sigma-Aldrich (St. Louis, MO).

Trapping protocols and chemical modification

To measure the state dependence of cysteine trapping in the desensitized state, we determined the baseline activation by 10 mM glutamate in the presence of 5 mM dithiothreitol (DTT), followed by the application of Cu:Phen (10 μM; prepared as described in (22)) and 100 μM Glu for a range of time intervals. To examine resting state trapping, we applied 10 μM Cu:Phen without agonist, and for some experiments, we added 100 μM CTZ. Each trapping exposure was delivered from the third barrel of the perfusion tool. For all trapping experiments, we quantified the relief of trapping by determining the fraction activated by sequential applications of 10 mM glutamate in 5 mM DTT immediately after trapping, as previously described (18). The envelope of the peak current responses after application of Cu:Phen were fit with a single exponential. By back extrapolating to the end of the Cu:Phen application, we were able to estimate the proportion of receptors that were trapped (22). The amplitude of the fit function was the trapped fraction of receptors, and we subtracted this fraction from 1 to get the active fraction (AF). For metal bridging experiments, Zn^{2+} was added (10 μM) to the external solution. To achieve zinc-free conditions, we added 10 μM EDTA, a potent Zn^{2+} chelator ($K_{D\ Zn^{2+}} = 10^{-16.4}$ M), to the external solution. We used the same analysis to determine the AF. We applied drugs to outside patches via perfusion tools made from custom manufactured glass tubing with four parallel barrels (VitroCom, Mountain Lakes, NJ) as described in (18). The glass was pulled to a final width of 200 μm, and the tip of the tool was etched in hydrofluoric acid and mounted in a piezo electric lever and controlled via a 100 V amplifier. The command voltage was filtered at 100 Hz to reduce vibration of the tool. When we measured the junction potential, the typical 10–90% rise time was 300 μs. For the fast oxidizing experiments, we determined the time that the patch spent in the Cu:Phen condition (third barrel) by measuring open tip currents, calibrating a voltage ramp protocol with different slopes to vary the dwell time in the third barrel from 5 to 30 ms (Fig. 7 *A*). We measured concentration-response curves for WT and the mutant A665W. We obtained the half-maximal effective concentration (EC_{50}) from fits to the Hill equation:

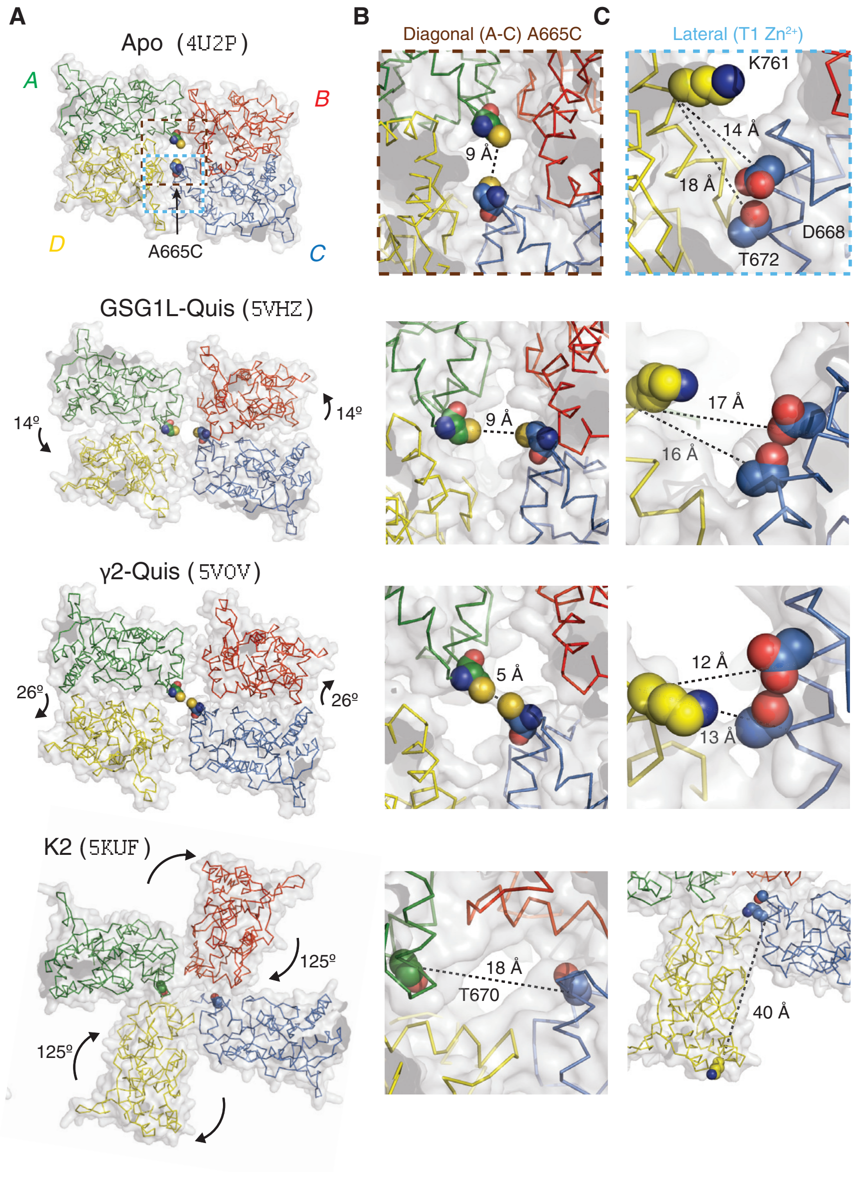

FIGURE 1 Candidate structures of glutamate receptors in desensitized states. (*A*) Plan views of the LBD layer in apo (13), 2xGSG1L Quis (16), γ-2 Quis (17), and GluK2 (2S,4R-4-methylglutamate (14) structures. PDB codes shown in brackets. Conformational changes relative to resting/active structures are indicated. Modeled A665C residues are shown as spheres. The scope of insets in (*B*) and (*C*) is shown by brown and light blue dashed boxes, respectively. (*B*) The A665C mutant site in the A and C subunits. Distances are modeled between cysteine sulfur atoms, except for GluK2 in which the equivalent residue is T670, which is buried. (*C*) The lateral interface between subunits C and D at the site of the zinc bridge mutant T1. Distances are measured between main chain C-α atoms. Nearest match residues for the T1 site in K2 are S669, K673, and K759. To see this figure in color, go online.

$$\frac{I}{I_{max}} = \frac{[A]^n}{[A]^n + [EC_{50}]^n}, \qquad (1)$$

where n is the Hill coefficient, $I_{\max}$ is the maximum response, and $[A]$ stands for the agonist concentration. Recovery from desensitization for the A665W mutant was measured with a 400-ms conditioning pulse.

Recovery from desensitization

To examine the recovery from desensitization for WT GluA2, patches containing hundreds of receptors were conditioned with applications of 10 mM glutamate for 50, 200, and 800 ms and 5 s. A test glutamate pulse was delivered at 12 different time points between 2 and 790 ms after the conditioning pulse. The protocols with different durations of the first pulse were randomly initiated for each patch and repeated if the patch was stable enough.

The timing and amplitude of peak currents and rise times of all peaks were measured in AxoGraph. Recordings from 23 patches from different cells were used for further analysis, except for the 5-s conditioning pulses. Because the duration of these measurements was long and the rundown of the current was often substantial, only measurements from four patches could be completed before the patch was lost and had sufficiently good quality for the whole set of records, namely 10–90% rise times of the glutamate response <500 μs and a stable baseline with fluctuations less than 10% of the peak current.

The response after a long (5 s) conditioning pulse was corrected for the slow rundown of current amplitudes caused by either accumulation of receptors into electrically isolated parts of the patch (23) or accumulation of receptors into nonfunctional states. A linear function was fitted to the currents and times of the response to the conditioning (first) pulse from each episode to extrapolate the expected maximum response at the time of each test (second) pulse. For each interval, the normalized responses were averaged, and the SD was used for fitting in IGOR Pro (WaveMetrics).

We and others have previously fitted recovery from desensitization with a Hodgkin-Huxley-type recovery curve (24):

$$f(t) = y_0 + (y_{max} - y_0) \cdot (1 - exp(-kt))^m, \qquad (2)$$

where k is the rate of recovery, m is the slope, y_{max} and y_0 are the maximum and minimum, respectively, and t is the interval between pulses. We also did this here for the data in Fig. 3 *A*. However, for the recovery after conditioning pulses of longer durations (Fig. 5), this function was insufficient because it could not describe the intermediate phases of recovery. These data could only be well fit by a function that was the sum of two Hodgkin-Huxley terms (with rates k_1 and k_2, slopes m_1 and m_2):

$$f(t) = y_0 + a_1 \cdot (1 - exp(-k_1 t))^{m_1} + (y_{max} - a_1 - y_0) \cdot (1 - exp(-k_2 t))^{m_2}. \quad (3)$$

To establish how unique the description by this two-component H.-H. function was, we tried several other different fit functions (three component H.-H. function, sigmoid, Hill) and varied the fit parameters (see Figs. S1 and S2; Tables S1 and S2). Time constants where quoted are reciprocals of rate constants.

Statistical analysis

All p-values were determined by a two-sample unpaired Student's t-test. The spread of the data where indicated is the SD of the mean.

Structural analysis and figure preparation was done in PyMOL (version 2.0; Schroedinger).

RESULTS

An interdimer interface forms in a desensitized state

To place intersubunit bridges in the context of the conformational changes that drive desensitization, we first compared the LBD layers of the apo state structure of GluA2 (Protein Data Bank, PDB: 4U2P (13)) to three candidate-desensitized state structures. We modeled the A665C mutation into these structures and measured the putative distances between the sulfur atoms of the cysteines. In the resting ligand-free structure, the SG-SG distance is 9 Å, partly because of the open angle adopted by the dimers but mainly as a consequence of the distance of the A and C subunits from the central axis of the receptor and ion pore. The structure of the GluA2 receptor in complex with the high-affinity full agonist L-quisqualate and the accessory subunit GSG1L (GluA2-2xGSG1LQuis. PDB: 5VHZ (16)) presents LBD dimers with broken local twofold symmetry, with disrupted interfaces between the dimers due to a rotation of 31° and translation of 6 Å of the upper D1 lobes. Despite these motions, the closest approach of modeled SG-SG distance remains 9 Å (Fig. 1). For comparison, in the active state structure PDB: 5WEO (25), the distance between cysteine sulfur atoms modeled at residue 665 is 11 Å. The GluA2-TARP γ-2 Quis structure (PDB: 5VOV (17)) shows similar rotations and translations within each of its LBD dimers. Placing the A665C mutations in subunits A and C of this structure allows the SG groups to be only 5 Å distant, within striking distance of forming a disulfide bridge, suggesting the TARP-bound desensitized state is quite compact; this concept was discussed at length in our previous work (21). A yet more dramatic change can be observed in the homologous GluK2 structure in the desensitized state (2S,4R-4-methylglutamate PDB: 5KUF (14)), which shows a rotation of the subunits D and B of 125°, producing pseudo four-fold symmetric arrangement of the LBDs. Similar large-scale disruptions of the LBD layer were observed in single particle analyses of GluA2 without auxiliary subunits (12–14). As a consequence of this movement, the residue T670 (equivalent in GluK2 to A665 in GluA2) is buried between the new interfaces formed between adjacent subunits (Fig. 1). For the same four candidate structures, we measured the distances between the residues that form the site of the T1 lateral Zn^{2+} bridge (19) built from introduced histidines (D668H T672H K761H; Fig. 1 *C*). The intersubunit distances are too great to predict zinc bridging for any of the candidate structures. The closest approach was for GluA2-TARP γ-2 Quis (PDB: 5VOV), in which the CA-CA distances were 12–13 Å (for K761 to D668 or T672). Overall, no candidate-desensitized arrangement would support a Zn^{2+} bridge if histidine residues were placed at these positions.

We reasoned that if we attempted to cross-link the diagonal A-C interface during desensitization, we could determine at which point in the desensitization reaction (either early or late) that these two subunits come together. Likewise, we expected that the lateral interface should not be readily accessible to desensitized receptors, unless a spectrum of different desensitized states are sampled. We used well-characterized cysteine substitutions at three positions in the FG-loop (I664, A665, and V666) (18,20,21). Each mutant was tested for its cross-linking potential in 100 μM glutamate. This concentration was based on the concentration dependence of desensitization of the WT receptor, which reaches full desensitization at 100 μM (22), with minimal activation. We used three barrels of a quadruple barrel fast perfusion system that enabled the application of 100 μM glutamate in the presence of 10 μM Cu:Phen with <10 ms resolution. We observed a dramatic reduction of the current activated by glutamate 10 mM after exposure to oxidizing conditions for the mutants I664C, A665C, and V666C (Fig. 2 *A*). The untrapped, AF was measured for different intervals of exposure to oxidizing conditions. After ~30 s of oxidizing conditions, the reduction of the AF reached a plateau for I664C, A665C, and V666C (to 31 ± 4, 29 ± 4, and 22 ± 3%, respectively; Fig. 2 *B*). The maximum extent of inhibition for the three mutants was similar, but this similarity does not imply that the underlying mechanism is the same. Factors that influence the extent of trapping include side-chain angles, state stability, steric hindrance, and the geometry of the subunits. We do note that trapping around the loop between F and G helices is position dependent, with much a reduced trapping extent at position 662, for example (18). The kinetics of recovery after trapping indicate how stable this interface is in the desensitized state. The time constants of recovery after trapping for I664C, A665C, and V666C were 1.4 ± 0.2, 3.3 ± 0.6, and 1.2 ± 0.4 s, respectively, after 10 s in Cu:Phen (Fig. 2 *C* and

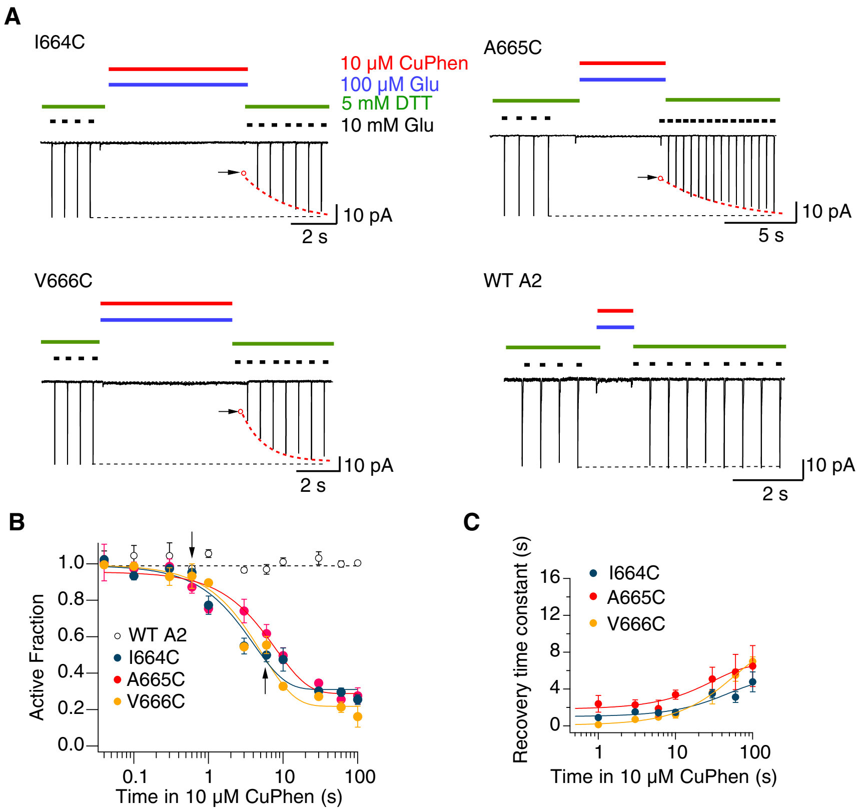

FIGURE 2 Contact between subunits A and C during desensitization. (*A*) Typical patch-clamp experiments for cysteine mutations at positions 664–666 and WT GluA2. Dotted red line is the exponential fit to recovery of the current in 10 mM glutamate and DTT, following trapping in the presence of 10 μM Cu:Phen and 100 μM glutamate. Open circles with an arrow indicate the presumptive back-extrapolated response immediately after trapping. (*B*) The AF of receptors after oxidization in the desensitized state plotted against the trapping interval (continuous lines are exponential fits). The probabilities of no difference in the AF after 10-s trapping in 10 μM Cu:Phen were 0.001, 0.0005, and 0.00003 for I664C, A665C, and V666C, respectively (compared to WT A2, Student's *t*-test, $n = 3$–4 patches per point). The arrows indicate the relevant intervals for the traces in (*A*). (*C*) The time constant of recovery after trapping is plotted against the trapping interval for I664C, A665C, and V666C, showing a positive correlation (continuous lines are exponential fits; $n = 3$–4 patches per point). To see this figure in color, go online.

see Discussion for details of the interpretation of these time constants). Strikingly, with the longer exposure to Cu:Phen, the three mutants I664C, A665C, and V666C each showed increased stabilization of the trapped state. We fitted the increase in the recovery time constant against exposure time with a single exponential to obtain the asymptotic maximum recovery time constants of 43 ± 13, 38 ± 19, and 43 ± 5 s for I664C, A665C, and V666C, respectively (Fig. 2 *C*). These results indicate that the A-C interface can be trapped in at least two desensitized states. We cannot discern from these data whether the states are accessed in parallel or series. One state recovers rapidly, but with long exposures, there is a progressive adoption of a state or set of trapped states that are considerably more stable.

A point mutant at the A-C interface slows recovery from desensitization

According to previous reports, the introduction of a cysteine in position A665 alters recovery from desensitization in a redox-sensitive manner (26). We hypothesized that the introduction of a bulky residue like tryptophan in position A665 should alter recovery if this interface forms during the desensitization process. To test this, we conditioned patches with an application of glutamate (10 mM for 100 ms), and then, a second application was delivered at varying times after the conditioning pulse. In these experiments, WT GluA2 recovered from desensitization with a time constant of 20 ms, as previously reported (27) In contrast, we observed a dramatic delay of desensitization recovery of more than sevenfold for the mutant A665W ($\tau_{rec} = 155 \pm 5$ ms; Fig. 3 *A*). The activation of the mutant and rate of entry to desensitization was indistinguishable from WT GluA2. We constructed a dose response curve for glutamate and observed little difference in the apparent affinity for glutamate (A665W $EC_{50} = 510 \pm 130$ μM compared to WT $EC_{50} = 330 \pm 100$ μM, with a *p*-value of no difference 0.19) (Fig. 3 *B*). We therefore ruled out the possibility that the change in recovery was due to an increase in affinity for glutamate in the A665W mutant. This observation further supports the idea that this intersubunit interface forms during the entry to or exit from desensitization.

Adoption of deep desensitized states protect against cross-linking

We reasoned that if the stable disulfide trapping we detected were unique to the desensitized state, then promoting desensitization should promote trapping and/or slow recovery. However, additional desensitized states might exist that are not readily disulfide linked by cysteines at the A-C interface. Such states would perhaps resemble the four-fold symmetry of the GluK2 structure or, more generally, would stably hinder the approach of cysteines at the otherwise proximal A-C interface because of a substantial conformational change. We tested the formation of intersubunit cross-links in a

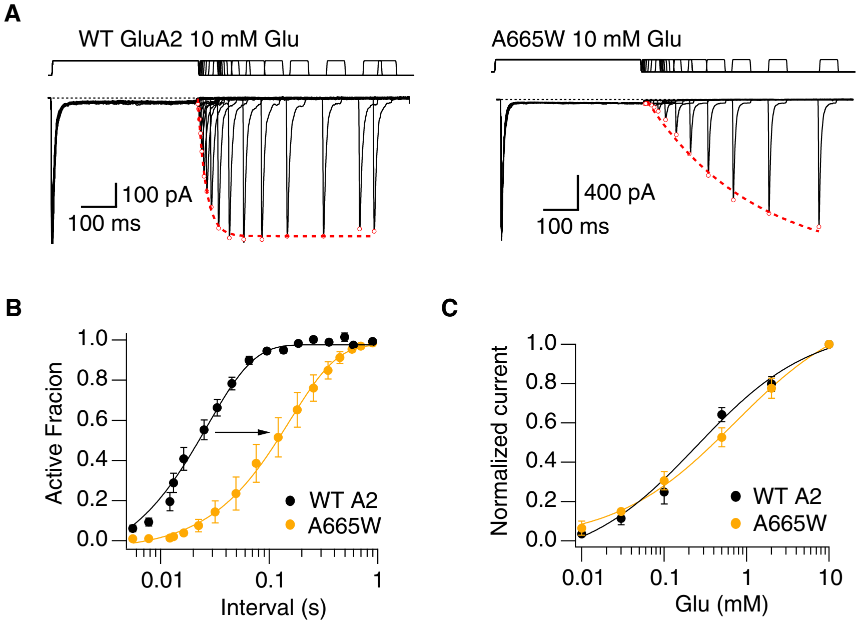

FIGURE 3 The A665W mutation slows down recovery from desensitization. (*A*) Currents evoked by 10 mM glutamate from WT GluA2 (*left panel*) and the mutant A665W (*right panel*). The two-pulse protocol had a conditioning pulse of 400 ms, followed by a second pulse at increasing intervals (responses are overlaid). Peak currents (*red open circles*) were fit with Hodgkin-Huxley functions (*dashed red lines*). (*B*) Mean of recovery from desensitization for GluA2 WT (*black*) and A665W (*orange*). For each interval, the peak of the second pulse is plotted as the AF (relative to the first peak) and fit with a Hodgkin-Huxley equation (with slope fixed to 2, see Materials and Methods). The time constants of recovery from desensitization were 20 $\pm$ 2 and 155 $\pm$ 5 ms for GluA2 WT and A665W, respectively (probability of no difference = 0.01, Student's *t*-test; $n = 4$). (*C*) Concentration-response curves for WT GluA2 (*blue circles*; $EC_{50} = 330 \pm 100$ μM) and GluA2 A665W (*yellow circles*; $EC_{50} = 510 \pm 130$ μM). The probability of no difference between the EC_{50} values was 0.19 ($n = 3$ cells). To see this figure in color, go online.

putative deep desensitized state using a mutant that strongly stabilizes the desensitized state (E713T and Y768R), with a recovery time constant of about 1 s (27). When we introduced a cysteine in position A665 in the single mutants E713T and Y768R, we observed less profound trapping than for the A665C mutant alone, with a reduction of the AF of 46% for A665C and E713T and 42% for A665C and Y768R after 100 s of application of Cu:Phen (Fig. 4, *A* and *B*). Both mutants showed slower recovery after trapping for A665C and E713T of 10.7 $\pm$ 1.7 and 9.7 $\pm$ 1.6 s for A665C and Y768R (Fig. 4 *C*). Again, longer exposures to Cu:Phen drove adoption of a yet more stable trapped arrangement. Fitting a single exponential to the recovery time constants versus the time in oxidizing conditions, we determined the asymptotic limiting time constants of a recovery of 27 $\pm$ 10 and 6 $\pm$ 1 s (for A665C, E713T, and A665C, Y768R, respectively) (Fig. 4 *C*). In dramatic contrast to these results, the triple mutant A665C ET/YR showed absolutely complete protection from trapping, exhibiting a similar profile to the WT GluA2 ET/YR mutant (Fig. 4 *A*). This observation suggests that this GluA2 mutant, which exhibits very stable desensitization, can adopt yet another deep-desensitized state in which the interface between subunits A and C is absent. Such a conformation is consistent with either the GluA2-2xGSG1LQuis structure or the GluK2 cryo-EM structure in which the equivalent residue for A665C is buried in intersubunit interfaces (Fig. 1).

Long exposures to glutamate promote entry to stable desensitized states

From these results, we predicted that the progressively greater stability of trapped receptors after long exposures to desensitized conditions should derive from the selective adoption of more stable desensitized states. However, the WT homomeric GluA2 receptor is known for its rapid and complete recovery from desensitization (see for example Fig. 3 *A*). To resolve this paradox, we tested if the rate of recovery from desensitization was sensitive to the duration of the conditioning pulse in two-pulse recovery experiments. In each record, a patch containing hundreds of WT GluA2 receptors was first conditioned with an application of glutamate (10 mM) for 50, 200, and 800 ms and 5 s. A second glutamate pulse was delivered at varying times after the conditioning pulse (2–790 ms; Fig. 5). For each patch, we made a series of recordings with the same conditioning pulse but different intervals and then repeated the protocol with a different conditioning pulse length. After short conditioning pulses, we observed prototypical fast recovery from desensitization for GluA2. The recovery after 50- and 200-ms conditioning pulses could be quite well described with a single H.-H.-type function with a time constant of 20 ms (with slope between 2.5 and 3; Fig. S1 *B*). However, in the same patch, a 5-s conditioning pulse of glutamate slowed the recovery profile in two distinct ways (Fig. 5 *E*). First, the fastest component, with a time constant of 14 ms, was only about 70% of the total amplitude (Table S1). Second, a small ($\sim$5%) but very slow ($\sim$1 s) component meant that recovery at the end of our protocol (700-ms interval) was always incomplete. Comparison of the recovery after 800-ms and 5-s conditioning pulses revealed both had a small intermediate component ($\sim$20%) with a time constant of about 50 ms (Table S1).

We also observed a slow component of recovery when using 10- or 30-s conditioning pulses, but it was hard to quantify because the recovery protocols using such long

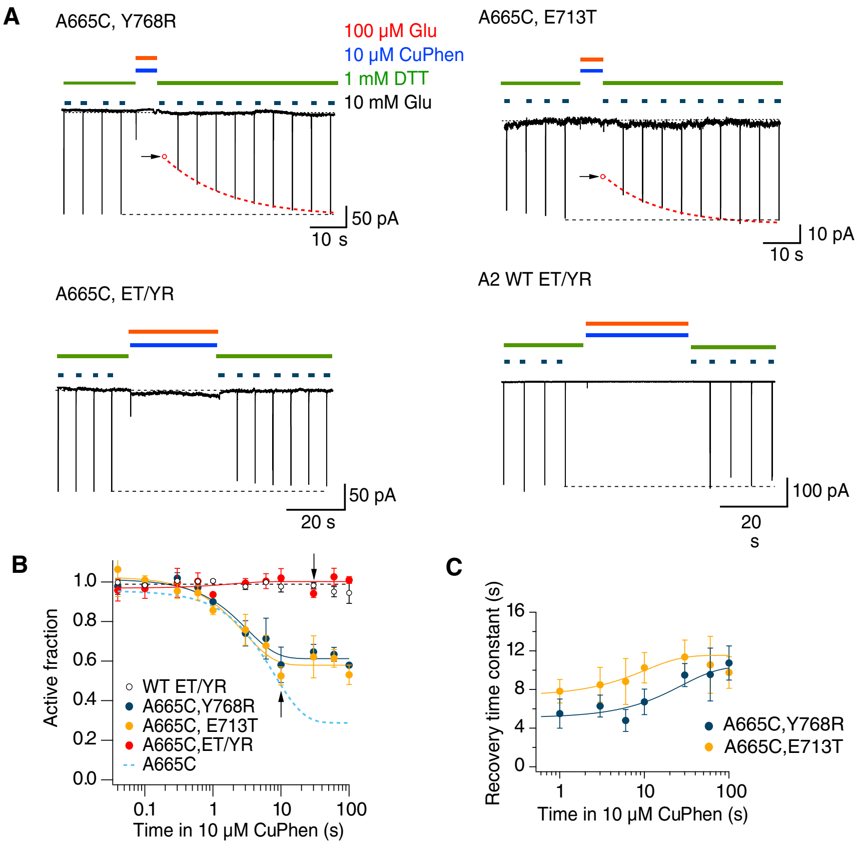

FIGURE 4 Protection of A665C in deep desensitized states. (*A*) Typical records show trapping and recovery for the mutants A665C Y768R (*upper left*) and A665C E713T (*upper right*). Arrows indicate the reduction of the current after trapping. The presumptive response (*open red circle*) was extrapolated from the double exponential fits to the recovery (*dashed red lines*). The mutant A665C on the ET/YR background (*lower left*) and the GluA2 ET/YR background (*lower right*) shows no modification. (*B*) The AF of receptors after oxidization in the desensitized state (continuous lines are exponential fits) is plotted against the trapping interval. The A665C trapping profile (*light blue dashed line*) and the fit to the ET/YR background (*black dashed line*) are indicated. The probability of no difference between the AF after 10 s of application of oxidizing conditions was 0.012 and 0.0008 for A665C and Y768R and A665C and E713T (versus A665C and ET/YR, Student's *t*-test, $n = 3$–4 patches per point). Arrows indicate intervals for the traces in (*A*). (*C*) Time constants of recovery after trapping plotted against the trapping interval for A665C, Y768R, A665C, and E713T (continuous lines are exponential fits, $n = 3$–4 patches per point). To see this figure in color, go online.

conditioning pulses necessarily lasted for 5–10 min, over which time even stable patches ran down and gave spurious responses. Taken together, these observations emphasize that WT GluA2 receptors can adopt a range of desensitized states with different stabilities, including very stable desensitized states.

Lateral shifts occur during desensitization

The GluA2-TARP γ2 Quis (PDB: 5VOV) structure (17) shows a compact packing of the lateral interface of the subunits A, B, C, and D of the LBDs, whereas in the GluA2-2xGSG1LQuis (PDB: 5VHZ) structure (16), this interface is clearly absent (Fig. 1 *C*). To analyze if this interface occurs in early desensitized states that can be adopted over millisecond timescales, we used a metal ion trapping approach. Previously, we engineered a pair of histidine mutants T1 (D668H, T672H, and K761H) and HH (D668H and K765H), which can coordinate Zn^{2+} between subunits A, B, C, and D at intermediate and high concentrations of glutamate, with CTZ present to block desensitization (19). Using these mutants, we detected the formation of the lateral interfaces in the desensitized state applying 100 μM glutamate in the presence of 10 μM Zn^{2+}. The mutants T1 and HH showed a reduction of the AF after 1 s of the application of Zn^{2+} in the desensitized state, with a reduction of the AF of 33% for T1 and 23% for HH plateauing after 100 s of Zn^{2+} application (Fig. 6 *A*), with time constants of recovery after trapping for T1 and HH of 1 ± 0.08 s, and 0.6 ± 0.08 s, respectively, after 10 s in zinc (Fig. 6 *B*). The asymptotic time constants of recovery in the limit of long Zn^{2+} exposures were 4 ± 0.6 and 2 ± 0.8 s (for T1 and HH, respectively; Fig. 6 *C*).

We hypothesized that the conformation trapped by the T1 lateral bridge is absent in the deep desensitized states promoted by the ET/YR mutation. To test this hypothesis, we inserted the triple histidine mutant T1 (D668H, T672H, and K761H) in the mutant (E713T and Y768R) background and tested its sensitivity to zinc in desensitized states. As for the A665C mutant, we did not detect the formation of T1 lateral interfaces in the presence of the ET/YR mutation. The T1 ET/YR mutant was as insensitive to 10- and 100-s application of 100 μM glutamate in the presence of 10 μM Zn^{2+} as WT GluA2 (Fig. 6 *A*). Double and single histidine mutant controls were also unaffected by Zn^{2+} application (Fig. 6 *B*), confirming the specificity of the trapping of the mutants T1 and HH. These experiments show that lateral shifts occur during desensitization, and the fast formation of bridges over tens of milliseconds suggests that these shifts occur during the first steps of the desensitization pathway. Additionally, we observed no lateral interface formation in the deep desensitized state.

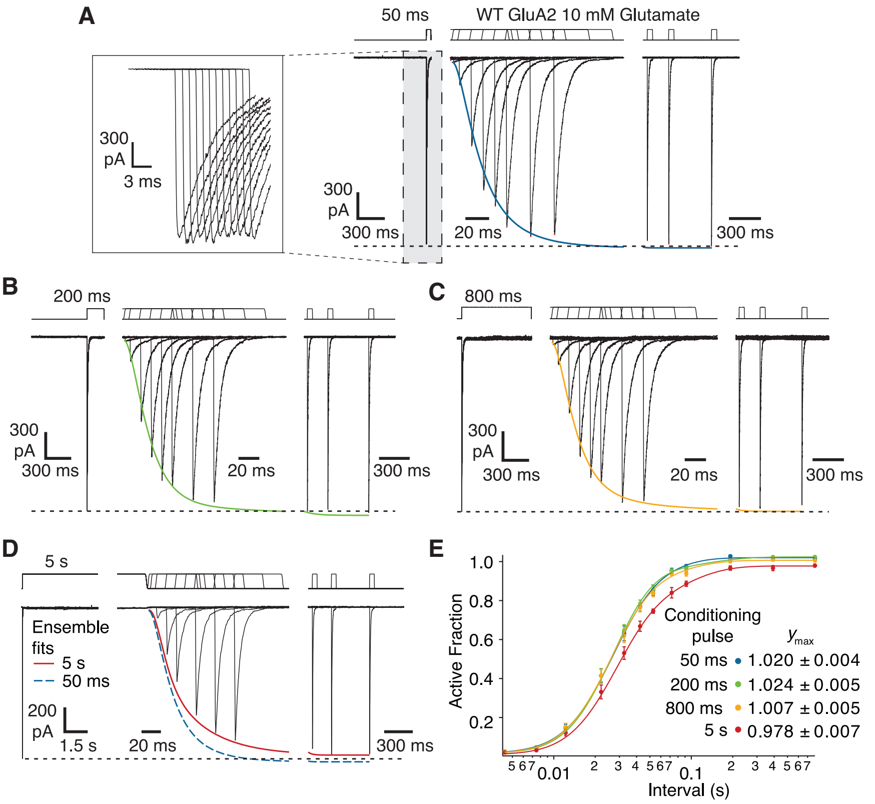

FIGURE 5 Recovery of WT GluA2. (*A–D*) Two-pulse protocols with conditioning pulses of 50 ms to 5 s. Inset (from *gray boxed area*) shows the typical consistency of responses to the conditioning pulse; responses were also highly consistent in their timing but are drawn displaced here. Ensemble fits to data with the two component H.-H. function (Eq. 3, see Materials and Methods). Conditioning pulses of 50 ms *blue*, 200 ms *green*, 800 ms *orange*, and 5 s *red* are overlaid on traces from immediate and late recovery responses (note changes of abscissa scale). Dashed black line indicates the responses to the conditioning pulse. In (*D*), the 50-ms ensemble fit is included as a dashed blue line. Note that the 5-s ensemble fit (*red*) did not reach the amplitude of the response to the conditioning pulse. The interval between episodes was typically 1 s. (*E*) Ensemble fits with Eq. 3 (H.-H. with two components). The slope of the fast component was fixed to 4, and the slope of the slower component was fixed to 2. Fitted maxima are shown. For the 5-s conditioning pulse, $a_1 = 0.7$, $k_1 = 71$ s^{-1} and $a_2 = 0.27$, $k_2 = 19$ s^{-1} with maximum 0.978 as shown. Alternative fits and fit parameters with errors are shown in Figs. S1 and S2 and Tables S1 and S2. To see this figure in color, go online.

Resting state desensitization is rapid and reversible

The differences between the NW-bound, putative desensitized structure (PDB: 4U4F) (28), the apo structure (13), and the resting-like state bound by the bulky competitive antagonist MPQX (29,30) are subtle, with little to no change in the distance between subunits A and C at A665C between 7 and 9 Å. All these structures have preserved intradimer active D1-D1 interfaces, and previous work showed that unbound and singly bound receptors can undergo desensitized transitions (22,31). This raises the question as to whether resting receptors can be trapped at the lateral interface as they desensitize. To investigate this point, we studied the formation of an interdimer cross-link between the residues A665C in the absence of the ligand. Initial experiments suggested that cross-linking was effectively instantaneous, using our regular protocols. To make the briefest application of Cu:Phen possible, we programmed a ramp stimulation for the perfusion tool, as illustrated in Fig. 7 *A*. With this protocol, we could apply oxidizing conditions for less than 5 ms. Exposing the mutant A665C to oxidizing conditions (10 μM Cu:Phen) in the absence of any agonist, we observed trapping of the A665C mutant that developed over ~10 ms, reducing the fraction of active receptors by ~20% (Fig. 7 *B*). This trapping was comfortably the fastest that we have observed in the AMPA receptor (18–20). To investigate whether this trapping requires breaking of the active dimer interface, we constrained the interface by exposing patches to 100 μM CTZ. There was a ~1000-fold delay in the formation of the diagonal disulfide between subunits A and C in resting state in the presence of CTZ, with a reduction of the AF of 20% only after a 100-s application of Cu:Phen (Fig. 7, *C* and *D*). The time constant of recovery after trapping in resting conditions was 380 ± 150 ms, but for resting + CTZ, the recovery was much faster (30 ± 5 ms) (after 10 s in Cu:Phen). These results show that the receptor transits between active (D1 intact) and desensitized-like (D1 broken) states on a more rapid timescale at rest than previously thought (22).

DISCUSSION

The idea that the interaction of the neurotransmitters with receptors encompasses more than a simple binding interaction followed by activation was a major step in receptor theory (32). It is now known that receptors in the brain have multiple active and inactive "desensitized" states. For example, the acetylcholine receptor presents at least four desensitized states (33), whereas for the BK potassium channel, which has multiple Ca^{2+} binding sites, desensitized configurations could represent up to 120 different states (34). Previous work has emphasized that native AMPA receptors, likely in complex with auxiliary subunits, have multiple desensitized states (2,35) as do TARP-associated

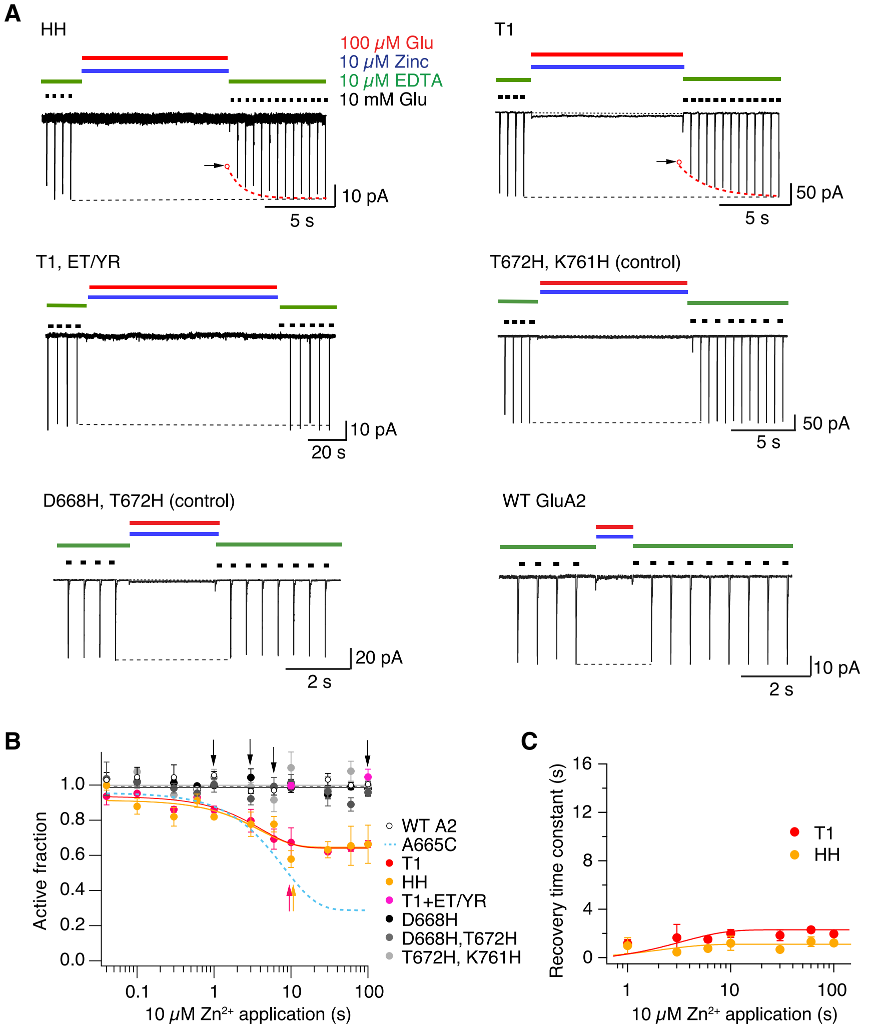

FIGURE 6 Lateral movements of the LBDs during desensitization. (*A*) Typical records for trapping and recovery from zinc trapping for the mutants HH (*upper left panel*) and T1 (*upper right*). Arrows indicate reduction of the current after trapping; dotted lines are double exponential fits to the recovery after trapping. The mutant T1 ET/YR shows no inhibition after 100-s exposure to zinc. Controls, such as the double mutants D668H and T672H and T672H and K761H, also showed no modification, behaving like WT GluA2. (*B*) The AF of receptors after Zn^{2+} exposure in the desensitized state (*continuous lines* are exponential fits) plotted against the interval. The probability of no difference (Student's *t*-test) to the WT A2 (*open circles*) after 10 s of Zn^{2+} was 0.018 for T1 (*red circles*) and 0.001 for HH (*yellow circles*). Controls and T1 ET/YR were indistinguishable from WT A2. Arrows indicate intervals for the traces in *A* (n = 3–4 patches per point). (*C*) The time constants of recovery after Zn^{2+} trapping plotted against the trapping interval for T1 and HH (n = 3–4 patches per point). To see this figure in color, go online.

receptors expressed recombinantly (36). For GluA2, auxiliary subunits γ-4 and γ-8 and CKAMP44 slow recovery from desensitization, whereas TARPs γ-2 and γ-3 have little effect (37–39). Multiple components in the recovery of AMPA receptors expressed in cell lines are detectable but less pronounced (31,40). However, the GluA2 (Q) homomer that we worked on here, and for which the majority of structural studies were completed, was until now widely reported to have a rapid and monotonic recovery from desensitization (see Fig. 3) (27,41,42).

We mapped inactive conformations of GluA2 over timescales from milliseconds to minutes with metal bridges and disulfide bonds that trap transient intersubunit interactions. This approach facilitated the detection of three distinct classes of desensitized states with glutamate and a fast, inactive state at rest (Fig. 8 *A*). Recovery from trapping was done in zero glutamate and reducing or chelating conditions; these measurements are closest to recovery from desensitization, and the time constants obtained provide estimates of the stability of the cross-linked desensitized state and transitions out of it. In the same experiments, we measured the time constants for the onset of the trapped states in steady 100 μM glutamate. Although in these conditions receptors are predominantly desensitized, these estimates involve receptors cycling through the desensitized and possibly other states.

We observed a positive correlation between the time of application of oxidizing conditions and the time constant of recovery after trapping for disulfide bonds at the A-C subunit interface and lateral zinc bridges. The simplest explanation is that prolonged exposure drives entry to at least two desensitized states, and the least frequently accessed are the most stable (see Fig. 8 *A*). The recovery from trapping in these desensitized states was dramatically slower than for the same mutants trapped in active states (18,19). Most surprisingly, by favoring slow recovery by introducing mutations in the D2 lobe of the LBDs, we could access a further, conformationally distinct, stable desensitized state that was immune to cross-linking at the A-C interface and that may or may not be physiological. It is tempting to consider these three distinct states as progressively profound conformational changes in the LBD layer, with more stable

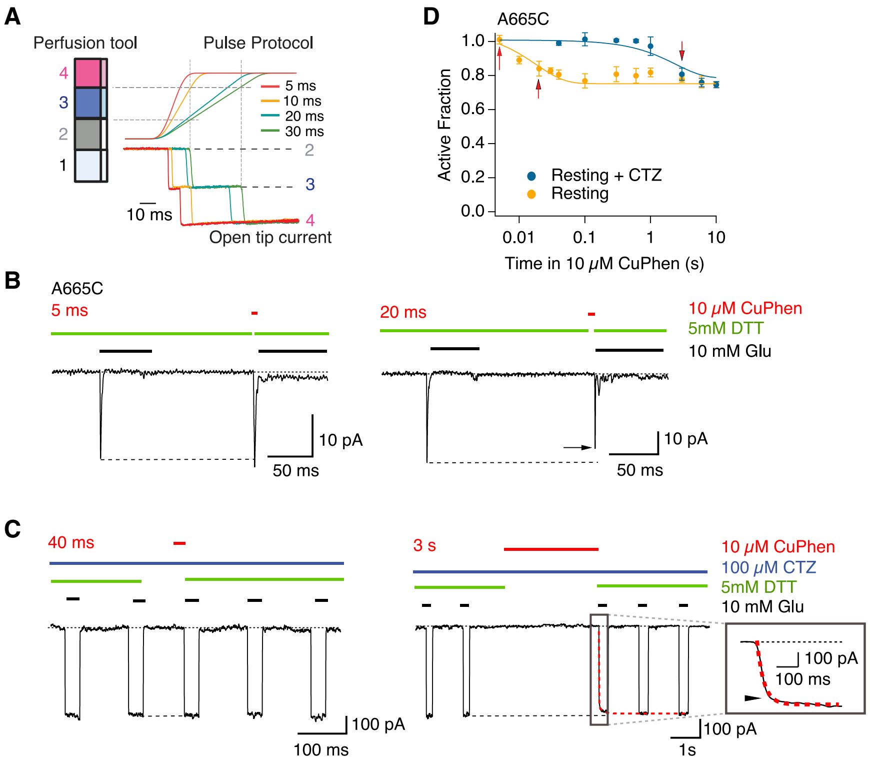

FIGURE 7 Rapid resting state desensitization. (*A*) Using the four-barrel fast perfusion system and switching from barrel 2 to 4, we applied oxidizing conditions for intervals as brief as 5 ms, with a voltage ramp command to the piezo lever (*right panel*). The open tip junction current shows that the pipette spends between 5 and 30 ms in the third barrel outflow (*blue*). Dashed lines indicate switch times for the 20-ms exposure. (*B*) Patch-clamp experiments showing Cu:Phen (10 μM) exposures of 5 ms (*left panel*) and 20 ms (*right panel*) for the mutant A665C in the resting state. Arrows indicate reduction of the current after modification. (*C*) Patch-clamp experiments show applications of Cu:Phen 10 μM for 40 ms (*left panel*) and 3 s (*right panel*) to. the mutant A665C in resting state with CTZ (100 μM). The inset shows the current profile during recovery; the arrow indicates reduction of the current after trapping. (*D*) The AF of receptors after oxidization in the desensitized state (*continuous lines*, exponential fit) is plotted against the trapping interval. Arrows indicate intervals for the traces in (*B* and *C*). The probability of no difference between the AF after trapping in resting state without and with CTZ for A665C after 10 s of application of oxidizing conditions was 0.003 (n = 3–6 patches per point). To see this figure in color, go online.

desensitized states corresponding to those seen in some structural biology experiments. In these experiments, ligand exposures are for technical reasons in minutes or hours. In Fig. 8 *B*, we outline a scheme to link the states identified from their cross-linking behavior to possible LBD arrangements. The conditions for cross-linking were specifically chosen to highly enrich desensitized conditions at the expense of resting and open receptors. However, we cannot be certain that our cross-links identify multiple desensitized states; they might instead act preferentially on transitions either into or out of desensitization. However, the mutants with more stable desensitized states slow down recovery from trapping, without slowing the adoption of deeply trapped states (Fig. 4)—the opposite of what would be expected if transitions alone were responsible for trapping. The detection of multiple time constants in the recovery of GluA2 WT, as predicted from the multiple states detected in our cross-linking data, provide good evidence against only transitions being involved in trapping.

Although cross-links do not define geometry uniquely, we note that they do report a minimum level of complexity in the conformational and dynamic space of GluA2. We identified three recovery time constants for WT GluA2 (~20, 100, and 1 s; from Fig. 5) that may correspond to the “fast,” “deep,” and “protected” classes (Fig. 8 *B*). Assuming this relation would imply that disulfides and zinc bridges trap these states ~100 times slower than the native states are entered (at the concentrations of Cu:Phen and Zn that we used) and extend the lifetime of the trapped states by ~100-fold. A back of the envelope calculation applying this logic to the observed time constants for the resting state trapping (entry ~10 ms, lifetime ~400 ms) would give the true resting state D1 dimer desensitization with a time constant for an entry of ~100 μs and a lifetime of ~4 ms. Providing some support for these estimates is the separate observation that trapping with bifunctional methanethiosulfonate (MTS) reagents can be accelerated 50-fold by simply increasing the reagent concentration (21). In this and other studies, we typically trap receptors in gentle (slow) conditions to reduce nonspecific cross-linking.

Even though prolonged oxidation can lead to promoting trapping by disulfides in stable inactive states, key weaknesses of this line of approach are that the presence of the bridges themselves could drive nonphysiological conformations, and the trapping bridges necessarily contribute to the lifetime of the trapped states. To address these points, we exposed WT GluA2 receptors to long applications of glutamate in two-pulse protocols and could detect slow components of recovery when we used conditioning pulses of 800 ms or longer. About one third of the population recovered either with an intermediate recovery rate ~4 times slower than the majority or on a timescale longer than the pulse protocol (>1 s). With the brief conditioning pulses that we and other investigators have routinely used (Fig. 3; (27,40,41)), these slow components are either very small or absent. GluA1 recovery could be fit by multiple single exponential functions (40). GluA2 has a steeper recovery profile than GluA1 and needs Hodgkin-Huxley type

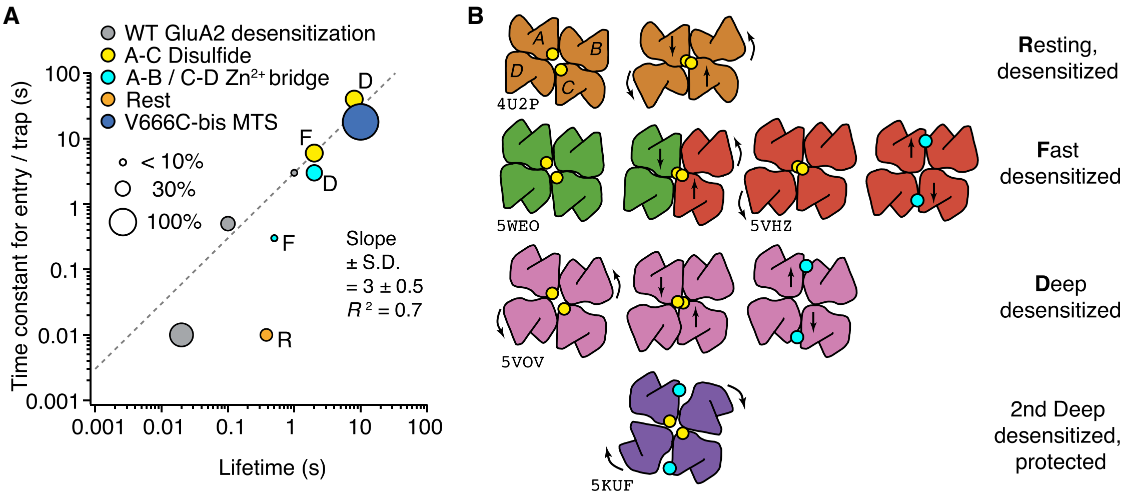

FIGURE 8 Models of trapping in desensitized states. (*A*) The time constants of trapping and entry to desensitization are plotted against desensitized or trapped state lifetimes. The symbol sizes represent the fraction of receptors trapped as indicated. Entry time constants depend on condition (zinc concentration, bis-MTS concentration), hence the linear relation has little physical meaning. Entry time constants for trapping were estimated from the midpoints of exponential fits to lifetimes. The time constants of entry, recovery, and the fractions for WT GluA2 are estimated from the data in Fig. 5 *E*. See the text for Discussion of these time constants and their putative relation to trapped states. Lettering indicates presumptive "R"esting, "F"ast, and "D"eep classes from (*B*). The V666C-bis-MTS point (*dark blue*) is from 1 μM bis-MTS trapping (21). (*B*) Putative LBD arrangements in four distinct classes of desensitized states were identified by cross-linking (plan view). Subunits A–D are arranged as marked on resting state. Published structures are indicated by PDB codes. Diagonal disulfide bonds are indicated by paired yellow circles, and the lateral Zn^{2+} bridge is indicated by a cyan circle (as in *A*). Fast desensitization can probably occur with one dimer active (*green*) and one dimer desensitized (*red*) after only small movements. Multiple components of trapping for both disulfides and zinc bridges require multiple states, here represented by the "Fast" and "Deep" desensitized rows (lettering from *A* indicated in *bold* here). In the second deep desensitized state, neither the A–C disulfide nor the lateral bridge can form. To see this figure in color, go online.

functions, usually fixed to have a slope exponent of 2. Intriguingly, a good description of the early phase of recovery required slope exponents >2 (see Figs. 5 and S1; Table S1). Fixing the slope to 4 offered a marginal improvement in the goodness of fit compared to a slope of 3. This observation is consistent with three or more independent particles being involved in the first recovery phase (24). A more qualitative observation is that, for conditioning pulses of 5 s or longer, we always observed rundown of the response. Although there are multiple explanations of rundown (see Materials and Methods), one source could be the irreversible accumulation of receptors into nonfunctional states. Quantitative measures of receptor conformation during such experiments (for example, from spectroscopy) may be able to provide information in this regard.

The desensitized state structure stabilized by GSG1L does not support the formation of the disulfide bond between A665C residues in subunits A and C (Fig. 1). This observation reinforces the idea that there are multiple desensitized states with common attributes but that some aspects of LBD geometry might be unimportant. Desensitized states in the AMPA receptor may correspond to any number of configurations in which the braced, active dimer arrangements are absent. Dissociation of a single active dimer is enough to desensitize the receptor (31). However, the overall configuration of the four LBDs might otherwise be compact. Auxiliary proteins with very different geometries (for example, TARPs and Shisa variants) seem to have distinct effects on the lifetime of the AMPA receptor desensitized state (43–45), and this could be because they stabilize different LBD arrangements in desensitized states. We cannot exclude the possibility that conformational changes could in principle bring the cysteines from subunits B and D (found on the outer flanks of the receptor in resting and activated states) into potential cross-linking positions. The rotations needed would be even more extreme than those reported for the kainate receptor. Likewise, for a zinc bridge to form diagonally (for example, between subunits B and D), an unprecedented lateral shift of the two LBD dimers would be needed. The requirement to avoid all simpler cross-linking geometries to reach such extreme states was previously discussed (21). Although experiments in heteromeric receptors (for example, GluA1:A2) could in principle provide more insight to subunit interactions, in our hands, cysteine cross-links bias heteromer assembly, and we could not so far generate zinc bridges at the diagonal A-C subunit interface.

Structures of GluA2 in the apo ligand free (13) and the MPQX-bound state (29,30) do not support a contact between subunits A and C in the resting state. Yet inactive states could be trapped by the A665C disulfide bond at "rest" within 10 ms. Therefore, these measurements place an upper limit of the timescale of latent rearrangements of the dimer interface. The S729C mutant forms a precedent for these observations (22), but for that mutant, resting state trapping was ~700-fold slower. Using CTZ, to stabilize the

D1-D1 interface, we could massively slow trapping in the resting state, suggesting that D1 dissociation is rapid and regular (46). It is likely that reformation of the interface at rest is at least as fast, otherwise the majority of receptors should simply desensitize upon binding glutamate. It is likely that the apo state is mobile and that the stability of the D1 dimer interface varies between flip and flop isoforms, impacting AMPA receptor kinetics (47). A second, less likely, effect of CTZ would be to reduce the conformational dynamics of the lower lobe of the LBD (where A665C is located) in the resting state. In the presence of partial agonists, twisting motions and other degrees of freedom have been reported (48–50), and these may be affected by CTZ binding. Even though the mutations used in this study (like the ET/YR mutant) were outside the D1 dimer interface, they could have their own effect on resting state dynamics. But any knock-on effect to alter cross-linking should be quite limited because the inefficiency of our cross-linking meant, on average, many cycles of receptor desensitization must occur before trapping, diluting any influence of resting state dynamics.

Our results are consistent with a previous report that shows differences in the rates of entry and recovery from desensitization in A665C receptors between oxidizing and reducing conditions (26). The introduction of tryptophan in position A665 produces a slow rate of recovery from desensitization of more than sevenfold. Therefore, a movement involving a close contact between subunits A and C at the loop between helices F and G of the LBDs appears necessary for fast recovery from desensitization.

Although the GluA2 homomeric receptor has been taken as the best structural model for synaptic receptors since the first structure was solved in 2009 (51), recent work and common sense suggests limitations in this regard. Whether or not receptors actually desensitize at most synapses remains controversial. Many synaptic receptors are likely triheteromers that include TARPs (52) and probably have richer desensitization behavior. These same limitations clearly apply to our work as well, which by its nature exploits a series of structures of the homomeric receptor.

In summary, our experiments provide insight into the conformations and kinetics of AMPA receptor desensitization. Particularly, this work suggests a hierarchy of AMPA receptor desensitized states. It seems likely that the more dispersed configurations of the extracellular domains detected in some structural biology experiments correspond to slowly attained states that may occur during brain injury or by receptors during biogenesis outside of fast excitatory synapses. However, compact desensitized arrangements of the LBD layer, probably like those stabilized by auxiliary proteins observed in cryo-EM experiments, are rapidly attained by AMPA receptors within milliseconds and on a timescale relevant for desensitization in the brain.

SUPPORTING MATERIAL

Supporting Material can be found online at https://doi.org/10.1016/j.bpj.2020.05.029.

AUTHOR CONTRIBUTIONS

All authors performed experiments, analyzed data, and wrote the article.

ACKNOWLEDGMENTS

We thank J. Baranovic for comments on the manuscript.

This work was supported by the European Research Council grant 647895 “GluActive”, the Deutsche Forschungsgemeinschaft (German Research Foundation) under Germany’s Excellence Strategy (EXC-2049 – 390688087), and the Deutsche Forschungsgemeinschaft grants PL619/1-1 and PL 619/3-1 (to A.J.R.P.). H.S. was the recipient of a Long-Term Fellowship from Human Frontier Science Program.

REFERENCES

1. Traynelis, S. F., L. P. Wollmuth, …, R. Dingledine. 2010. Glutamate receptor ion channels: structure, regulation, and function. *Pharmacol. Rev.* 62:405–496.
2. Colquhoun, D., P. Jonas, and B. Sakmann. 1992. Action of brief pulses of glutamate on AMPA/kainate receptors in patches from different neurones of rat hippocampal slices. *J. Physiol.* 458:261–287.
3. Robert, A., N. Armstrong, …, J. R. Howe. 2005. AMPA receptor binding cleft mutations that alter affinity, efficacy, and recovery from desensitization. *J. Neurosci.* 25:3752–3762.
4. Saviane, C., and R. A. Silver. 2006. Fast vesicle reloading and a large pool sustain high bandwidth transmission at a central synapse. *Nature.* 439:983–987.
5. Crowley, J. J., A. G. Carter, and W. G. Regehr. 2007. Fast vesicle replenishment and rapid recovery from desensitization at a single synaptic release site. *J. Neurosci.* 27:5448–5460.
6. Bowie, D. 2008. Ionotropic glutamate receptors & CNS disorders. *CNS Neurol. Disord. Drug Targets.* 7:129–143.
7. Christie, L. A., T. A. Russell, …, A. Contractor. 2010. AMPA receptor desensitization mutation results in severe developmental phenotypes and early postnatal lethality. *Proc. Natl. Acad. Sci. USA.* 107:9412–9417.
8. Penn, A. C., S. R. Williams, and I. H. Greger. 2008. Gating motions underlie AMPA receptor secretion from the endoplasmic reticulum. *EMBO J.* 27:3056–3068.
9. Priel, A., S. Selak, …, Y. Stern-Bach. 2006. Block of kainate receptor desensitization uncovers a key trafficking checkpoint. *Neuron.* 52:1037–1046.
10. Armstrong, N., and E. Gouaux. 2000. Mechanisms for activation and antagonism of an AMPA-sensitive glutamate receptor: crystal structures of the GluR2 ligand binding core. *Neuron.* 28:165–181.
11. Armstrong, N., J. Jasti, …, E. Gouaux. 2006. Measurement of conformational changes accompanying desensitization in an ionotropic glutamate receptor. *Cell.* 127:85–97.
12. Meyerson, J. R., J. Kumar, …, S. Subramaniam. 2014. Structural mechanism of glutamate receptor activation and desensitization. *Nature.* 514:328–334.
13. Dürr, K. L., L. Chen, …, E. Gouaux. 2014. Structure and dynamics of AMPA receptor GluA2 in resting, pre-open, and desensitized states. *Cell.* 158:778–792.

14. Meyerson, J. R., S. Chittori, …, S. Subramaniam. 2016. Structural basis of kainate subtype glutamate receptor desensitization. *Nature.* 537:567–571.

15. Plested, A. J. R., R. Vijayan, …, M. L. Mayer. 2008. Molecular basis of kainate receptor modulation by sodium. *Neuron.* 58:720–735.

16. Twomey, E. C., M. V. Yelshanskaya, …, A. I. Sobolevsky. 2017. Structural bases of desensitization in AMPA receptor-auxiliary subunit complexes. *Neuron.* 94:569–580.e5.

17. Chen, S., Y. Zhao, …, E. Gouaux. 2017. Activation and desensitization mechanism of AMPA receptor-TARP complex by cryo-EM. *Cell.* 170:1234–1246.e14.

18. Lau, A. Y., H. Salazar, …, B. Roux. 2013. A conformational intermediate in glutamate receptor activation. *Neuron.* 79:492–503.

19. Baranovic, J., M. Chebli, …, A. J. Plested. 2016. Dynamics of the ligand binding domain layer during AMPA receptor activation. *Biophys. J.* 110:896–911.

20. Salazar, H., C. Eibl, …, A. Plested. 2017. Mechanism of partial agonism in AMPA-type glutamate receptors. *Nat. Commun.* 8:14327.

21. Baranovic, J., and A. J. Plested. 2018. Auxiliary subunits keep AMPA receptors compact during activation and desensitization. *eLife.* 7:e40548.

22. Plested, A. J. R., and M. L. Mayer. 2009. AMPA receptor ligand binding domain mobility revealed by functional cross linking. *J. Neurosci.* 29:11912–11923.

23. Suchyna, T. M., V. S. Markin, and F. Sachs. 2009. Biophysics and structure of the patch and the gigaseal. *Biophys. J.* 97:738–747.

24. Hodgkin, A. L., and A. F. Huxley. 1952. A quantitative description of membrane current and its application to conduction and excitation in nerve. *J. Physiol.* 117:500–544.

25. Twomey, E. C., M. V. Yelshanskaya, …, A. I. Sobolevsky. 2017. Channel opening and gating mechanism in AMPA-subtype glutamate receptors. *Nature.* 549:60–65.

26. Yelshanskaya, M. V., K. Saotome, …, A. I. Sobolevsky. 2016. Probing intersubunit interfaces in AMPA-subtype ionotropic glutamate receptors. *Sci. Rep.* 6:19082.

27. Carbone, A. L., and A. J. R. Plested. 2012. Coupled control of desensitization and gating by the ligand binding domain of glutamate receptors. *Neuron.* 74:845–857.

28. Yelshanskaya, M. V., M. Li, and A. I. Sobolevsky. 2014. Structure of an agonist-bound ionotropic glutamate receptor. *Science.* 345:1070–1074.

29. Twomey, E. C., M. V. Yelshanskaya, …, A. I. Sobolevsky. 2016. Elucidation of AMPA receptor-stargazin complexes by cryo-electron microscopy. *Science.* 353:83–86.

30. Zhao, Y., S. Chen, …, E. Gouaux. 2016. Architecture of fully occupied GluA2 AMPA receptor-TARP complex elucidated by cryo-EM. *Nature.* 536:108–111.

31. Robert, A., and J. R. Howe. 2003. How AMPA receptor desensitization depends on receptor occupancy. *J. Neurosci.* 23:847–858.

32. Katz, B., and S. Thesleff. 1957. A study of the desensitization produced by acetylcholine at the motor end-plate. *J. Physiol.* 138:63–80.

33. Elenes, S., and A. Auerbach. 2002. Desensitization of diliganded mouse muscle nicotinic acetylcholine receptor channels. *J. Physiol.* 541:367–383.

34. Magleby, K. L. 2003. Gating mechanism of BK (Slo1) channels: so near, yet so far. *J. Gen. Physiol.* 121:81–96.

35. Patneau, D. K., and M. L. Mayer. 1991. Kinetic analysis of interactions between kainate and AMPA: evidence for activation of a single receptor in mouse hippocampal neurons. *Neuron.* 6:785–798.

36. Levchenko-Lambert, Y., D. M. Turetsky, and D. K. Patneau. 2011. Not all desensitizations are created equal: physiological evidence that AMPA receptor desensitization differs for kainate and glutamate. *J. Neurosci.* 31:9359–9367.

37. Cais, O., B. Herguedas, …, I. H. Greger. 2014. Mapping the interaction sites between AMPA receptors and TARPs reveals a role for the receptor N-terminal domain in channel gating. *Cell Rep.* 9:728–740.

38. von Engelhardt, J., V. Mack, …, H. Monyer. 2010. CKAMP44: a brain-specific protein attenuating short-term synaptic plasticity in the dentate gyrus. *Science.* 327:1518–1522.

39. Carbone, A. L., and A. J. R. Plested. 2016. Superactivation of AMPA receptors by auxiliary proteins. *Nat. Commun.* 7:10178.

40. Bowie, D., and G. D. Lange. 2002. Functional stoichiometry of glutamate receptor desensitization. *J. Neurosci.* 22:3392–3403.

41. Zhang, W., A. Robert, …, J. R. Howe. 2006. The relationship between agonist potency and AMPA receptor kinetics. *Biophys. J.* 91:1336–1346.

42. Weston, M. C., C. Gertler, …, C. Rosenmund. 2006. Interdomain interactions in AMPA and kainate receptors regulate affinity for glutamate. *J. Neurosci.* 26:7650–7658.

43. Priel, A., A. Kolleker, …, Y. Stern-Bach. 2005. Stargazin reduces desensitization and slows deactivation of the AMPA-type glutamate receptors. *J. Neurosci.* 25:2682–2686.

44. Farrow, P., K. Khodosevich, …, J. von Engelhardt. 2015. Auxiliary subunits of the CKAMP family differentially modulate AMPA receptor properties. *eLife.* 4:e09693.

45. Eibl, C., and A. J. R. Plested. 2018. AMPA receptors: mechanisms of auxiliary protein action. *Curr. Opin. Physiol.* 2:84–91.

46. Gonzalez, J., M. Du, …, V. Jayaraman. 2010. Role of dimer interface in activation and desensitization in AMPA receptors. *Proc. Natl. Acad. Sci. USA.* 107:9891–9896.

47. Dawe, G. B., M. F. Kadir, …, D. Bowie. 2019. Nanoscale mobility of the apo state and TARP stoichiometry dictate the gating behavior of alternatively spliced AMPA receptors. *Neuron.* 102:976–992.e5.

48. Fenwick, M. K., and R. E. Oswald. 2008. NMR spectroscopy of the ligand-binding core of ionotropic glutamate receptor 2 bound to 5-substituted willardiine partial agonists. *J. Mol. Biol.* 378:673–685.

49. Ahmed, A. H., S. Wang, …, R. E. Oswald. 2011. Mechanism of AMPA receptor activation by partial agonists: disulfide trapping of closed lobe conformations. *J. Biol. Chem.* 286:35257–35266.

50. Birdsey-Benson, A., A. Gill, …, D. R. Madden. 2010. Enhanced efficacy without further cleft closure: reevaluating twist as a source of agonist efficacy in AMPA receptors. *J. Neurosci.* 30:1463–1470.

51. Sobolevsky, A. I., M. P. Rosconi, and E. Gouaux. 2009. X-ray structure, symmetry and mechanism of an AMPA-subtype glutamate receptor. *Nature.* 462:745–756.

52. Zhao, Y., S. Chen, …, E. Gouaux. 2019. Architecture and subunit arrangement of native AMPA receptors elucidated by cryo-EM. *Science.* 364:355–362.

Biophysical Journal, Volume *119*

Supplemental Information

Measurements of the Timescale and Conformational Space of AMPA Receptor Desensitization

Hector Salazar, Sabrina Mischke, and Andrew J.R. Plested

Supplementary Table 1 Hodgkin-Huxley type fits to GluA2 recovery from desensitization with 1, 2 or 3 components.

Figure	Function	Duration of conditioning pulse	Goodness of fit (χ^2)	Parameters									
				y_0	a_1	a_2	k_1	k_2	k_3	m_1	m_2	m_3	y_{max}
S1A	H.-H. 1 component	50ms	44	0.000 ± 0.004			43 ± 1			*2*			1.022 ± 0.003
		200ms	36	0.000 ± 0.006			44 ± 1			*2*			1.022 ± 0.004
		800ms	41	0.000 ± 0.006			44 ± 1			*2*			1.007 ± 0.004
		5s	153	0.000 ± 0.002			34 ± 1			*2*			0.983 ± 0.006
S1B	H.-H. 1 component	50ms	13	0.010 ± 0.006			54 ± 3			2.8 ± 0.3			1.017 ± 0.003
		200ms	14	0.0011 ± 0.009			52 ± 3			2.6 ± 0.3			1.018 ± 0.004
		800ms	18	0.00 ± 0.01			52 ± 3			2.5 ± 0.3			1.001 ± 0.004
		5s	29	0.002 ± 0.006			46 ± 2			2.8 ± 0.2			0.967 ± 0.006
S1C	H.-H. 2 component	50ms	940	0.000 ± 0.009	0.5 ± 6000		21 ± 1000	21 ± 353		*1*	*1*		1.035 ± 0.004
		200ms	610	0.00 ± 0.01	0.6 ± 8000		24 ± 1000	24 ± 2000		*1*	*1*		1.033 ± 0.005
		800ms	610	0.00 ± 0.01	0.7 ± 6000		24 ± 1000	24 ± 3000		*1*	*1*		1.020 ± 0.005
		5s	2079	0.000 ± 0.006	0.5 ± 6000		12 ± 4000	12 ± 3000		*1*	*1*		1.02 ± 0.02
S1D	H.-H. 2 component	50ms	44	0.000 ± 0.006	0.79 ± 3000		43 ± 2000	42 ± 6000		*2*	*2*		1.022 ± 0.004
		200ms	36	0.000 ± 0.008	0.50 ± 8000		44 ± 8000	45 ± 7000		*2*	*2*		1.022 ± 0.004
		800ms	42	0.000 ± 0.009	1 ± 5		43 ± 38	30 ± 4000		*2*	*2*		1.006 ± 0.005
		5s	154	0.000 ± 0.005	0.6 ± 6000		34 ± 4000	33 ± 5000		*2*	*2*		0.984 ± 0.008
S1E	H.-H. 2 component	50ms	9	0.01 ± 0.005	0.9 ± 0.2		61 ± 4	20 ± 20		*3*	*2*		1.02 ± 0.004
		200ms	4	0.005 ± 0.006	0.96 ± 0.03		61 ± 3	10 ± 5		*3*	*2*		1.025 ± 0.006
		800ms	8	0.005 ± 0.006	0.91 ± 0.07		63 ± 4	15 ± 10		*3*	*2*		1.007 ± 0.004
		5s	14	0.0002 ± 0.004	0.84 ± 0.07		55 ± 3	14 ± 6		*3*	*2*		0.978 ± 0.007
S1F	H.-H. 3 component	50ms	39	0.017 ± 0.007	0.79 ± 0.19	0.2 ± 530	75 ± 5	30 ± 30000	60 ± 2000	*4*	*2*	*2*	
		200ms	34	0.01 ± 0.01	0.78 ± 0.25	0.1 ± 440	75 ± 13	33 ± 4000	31 ± 5000	*4*	*2*	*2*	
		800ms	6	0.016 ± 0.006	0.85 ± 0.08	0.16 ± 0.07	76 ± 4	16 ± 10	3 ± 3	*4*	*2*	*2*	
		5s	7	0.008 ± 0.005	0.69 ± 0.09	0.28 ± 0.09	71 ± 4	20 ± 6	1 ± 2	*4*	*2*	*2*	
5	H.-H. 2 component	50ms	6	0.015 ± 0.006	0.7 ± 0.2		76 ± 4	30 ± 8		*4*	*2*		1.020 ± 0.004
		200ms	3	0.016 ± 0.005	0.91 ± 0.04		73 ± 3	13 ± 5		*4*	*2*		1.024 ± 0.005
		800ms	7	0.015 ± 0.007	0.8 ± 0.1		77 ± 5	22 ± 9		*4*	*2*		1.007 ± 0.005
		5s	7	0.008 ± 0.004	0.70 ± 0.08		71 ± 4	19 ± 4		*4*	*2*		0.978 ± 0.007

Parameters are shown ± their standard deviation from the fit in IGOR Pro. Parameters in red are not properly determined, and those italicised were fixed. The goodness of fit (χ^2) is coloured from worst (red) to best (green). The two component H.-H. fit with exponents m_1 and m_2 fixed to 1 is equivalent to a double exponential fit. The 1 and 2 component H.-H. functions are given in the methods, main text . The H.-H. 3 Component function (maximum fixed to 1) is as follows:

$$f(t) = y_0 + a_1 \cdot (1 - exp(-k_1 t))^{m_1} + a_2 \cdot (1 - exp(-k_2 t))^{m_2} + (1 - a_1 - a_2 - y_0) \cdot (1 - exp(-k_3 t))^{m_3}$$

Supplementary Table 2 Sigmoid and Hill type fits to GluA2 recovery from desensitization

Figure	Function	Duration of conditioning pulse	Goodness of fit (χ^2)	Parameters				
				y_0	τ	m	y_{max}	t_{50}
S2A	Sigmoid	50ms	100	0.00 ± 0.01	0.0083 ± 0.0006		1.01 ± 0.01	0.0314 ± 0.0008
		200ms	104	0.00 ± 0.02	0.0081 ± 0.0007		1.01 ± 0.02	0.0305 ± 0.0009
		800ms	111	0.00 ± 0.02	0.0089 ± 0.0007		0.99 ± 0.02	0.0313 ± 0.0009
		5s	157	0.00 ± 0.01	0.0095 ± 0.0006		0.95 ± 0.01	0.0381 ± 0.0009
S2B	Hill	50ms	9	0.014 ± 0.004		2.5 ± 0.1	1.022 ± 0.004	0.0269 ± 0.0007
		200ms	5	0.009 ± 0.007		2.5 ± 0.1	1.022 ± 0.004	0.0265 ± 0.0009
		800ms	8	0.008 ± 0.008		2.4 ± 0.1	1.007 ± 0.004	0.0265 ± 0.0008
		5s	13	0.002 ± 0.005		2.4 ± 0.1	0.975 ± 0.006	0.0317 ± 0.0008

Parameters are shown ± their standard deviations from fits in IGOR Pro. The parameter y_0 was constrained to be greater than zero for the sigmoid fits. The goodness of fit (χ^2) is coloured from worst (red) to best (green) according to the scale in Supplementary Table 1.

Sigmoid function:

$$f(t) = y_0 + \left(y_{max} \middle/ \left(1 + exp\left(\frac{t_{50} - t}{\tau}\right)\right)\right)$$

Hill equation:

$$f(t) = y_0 + (y_{max} - y_0) \,/\, \left(1 + \left(\frac{t_{50}}{t}\right)^m\right)$$

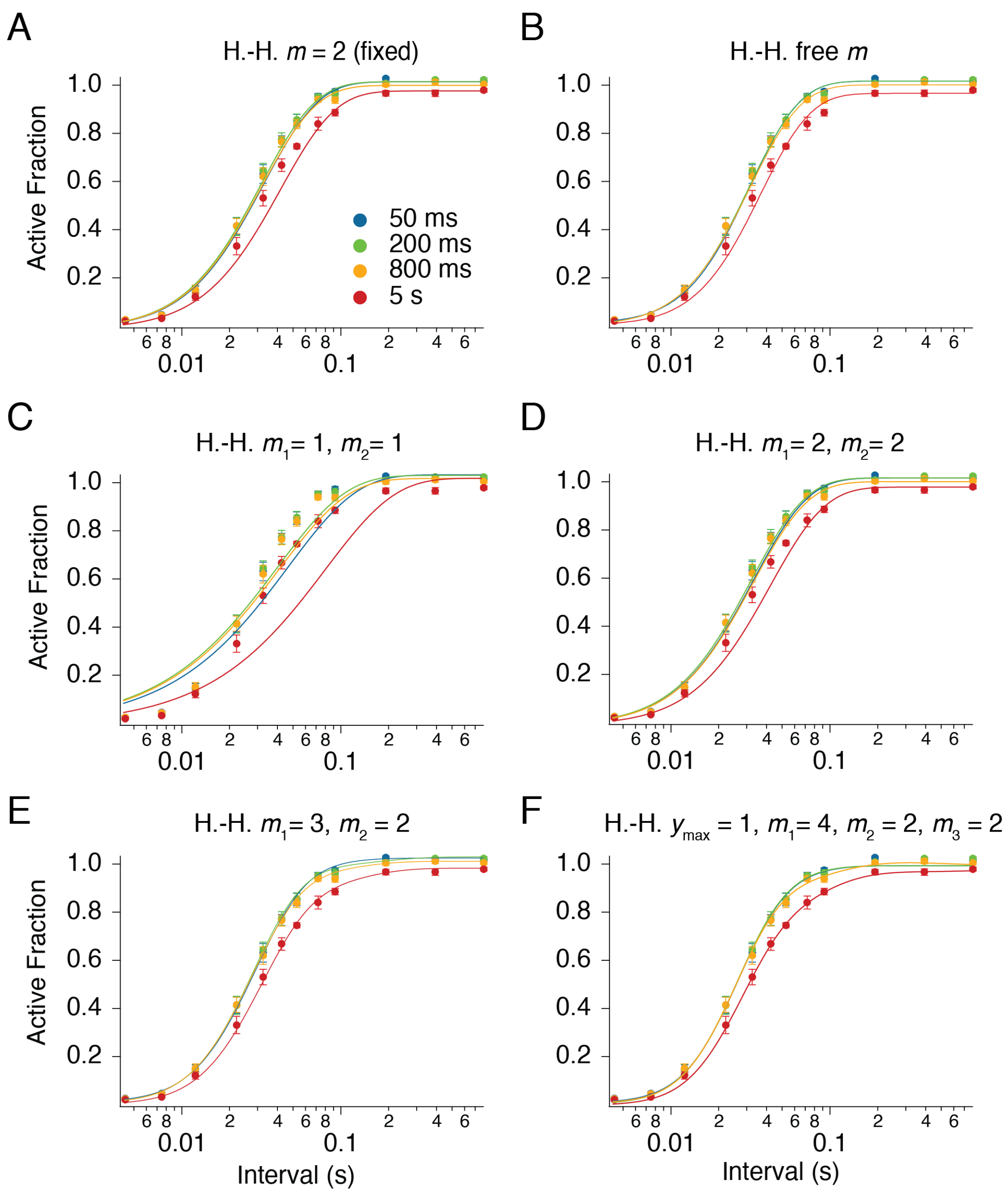

Supplementary Figure 1 Hodgkin-Huxley fits to GluA2 recovery from desensitization

Parameters and χ^2 values for each fitted curve are given in Supplementary Table 1. Data points from Figure 5.

(A) Single component H.-H. fit with slope fixed to 2 cannot describe the recovery following a 5 s pulse (red). The early phase of recovery is poorly captured by this function because it is not steep enough.

(B) Allowing the slope to vary in a single component H.-H. function makes a better description of the early phase, but not for the recovery following the 5 s conditioning pulse (red curve). The disadvantage of a free slope is that rates and slopes are strongly negatively correlated during the fitting process.

(C) A two component H.-H. function with slopes fixed to 1 (equivalent to a double exponential) is a very poor descriptor, because the slopes are too shallow. Parameters in this fit are undetermined.

(D) A two component H.-H. function with both slopes fixed to two gives a poor description of the data following a 5 s conditioning pulse. For the shorter pulse data, the parameters are not determined.

(E) With one slope fixed to 3 and one slope fixed to 2, the description is good and all parameters are well determined. The goodness of fit (χ^2) for each curve was similar to fits with $m_1 = 4$ and $m_2 = 2$ (Figure 5), except for recovery following the 5 s pulse, where the fit is definitively worse ($\chi^2 = 14$ vs. 7).

(F) The H.-H. function with three components (with fixed slopes, and maximum fixed to 1) gives a good description for recovery following the 5 s condition pulse. For shorter conditioning pulses, several parameters are not defined. For the recovery following the 800 ms pulse (orange curve), the slowest component ($k_3 = 3\ s^{-1}$) has a negative amplitude.

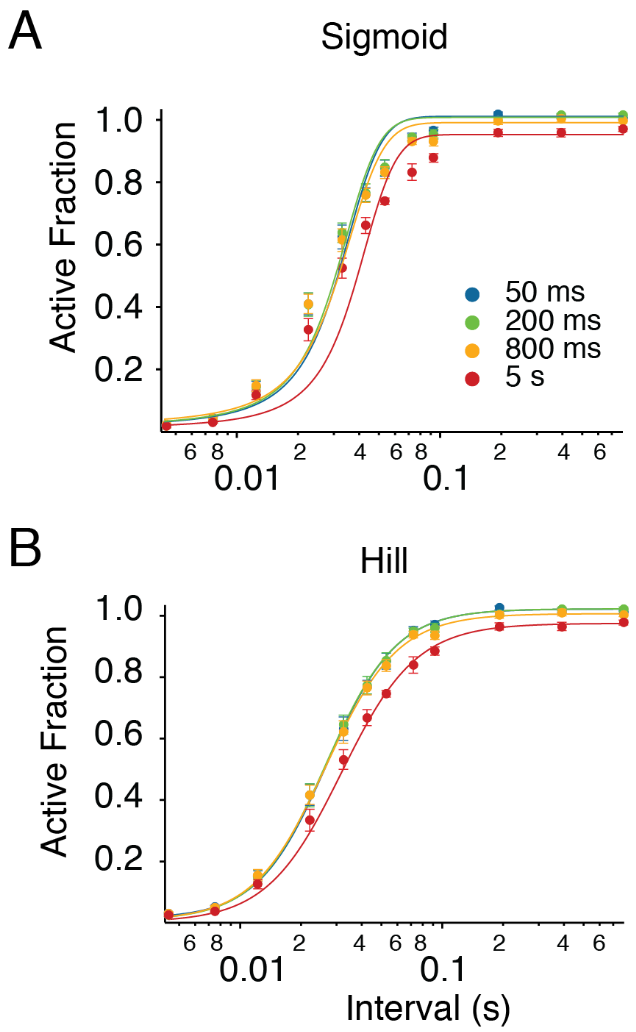

Supplementary Figure 2 Sigmoid and Hill fits to GluA2 recovery from desensitization
Equations, and parameters and χ^2 values for each fitted curve are given in Supplementary Table 2 and its legend.
(A) Fits of sigmoid curves are extremely poor at describing the recovery, because they are too steep. The y_0 parameter was fixed to be positive.
(B) Allowing a free slope in the Hill equation describes the recovery from desensitization data surprisingly well, almost as well as any other function. The slope of the early phase of recovery is poorly described for the recovery after the 5 s conditioning pulse (red curve). The disadvantage of a free slope is that rate and slope are strongly negatively correlated during the fitting process. Overall, fitting the data with the Hill relation is problematic because the physical meaning of the equation with relation to conformational changes over time is unclear.
